# Two tris­(3,5-disubstituted phen­yl)phosphines and their isostructural P^V^ oxides

**DOI:** 10.1107/S2056989018007831

**Published:** 2018-06-05

**Authors:** Nathan D. D. Hill, René T. Boeré

**Affiliations:** a Chemistry and Biochemistry, University of Lethbridge, 4401 University Drive West, Lethbridge, Alberta, T1K3M4, Canada

**Keywords:** crystal structure, phosphane, di­aryl­phospho­ryl­benzene, phosphine oxide, π-stacking, packing forces, conformation

## Abstract

The crystal structures of tris­(3,5-di­methyl­phen­yl)phosphine (C_24_H_27_P), (I), tris­(3,5-di­methyl­phen­yl)phosphine oxide (C_24_H_27_OP), (II), tris­(4-meth­oxy-3,5-di­methyl­phen­yl)phosphine (C_27_H_33_O_3_P), (III), and tris­(4-meth­oxy-3,5-di­methyl­phen­yl)phosphine oxide (C_27_H_33_O_4_P), (IV), are reported. The strucure of (III) has been described before [Romain *et al.* (2000 ▸). *Organometallics*, **19**, 2047–2050], but it is rereported here on the basis of modern area-detector data and to facilitate comparison with the other structures reported here. Compounds (I) and (II) crystallize isostructurally in *P*2_1_/*c*. Similarly, (III) and (IV) crystallize isostructurally in *Pbca*. The conformations of (I) and (II) in the solid state deviate strongly from helical, whereas those of (III) and (IV) are found to be closer to an ideal threefold rotational symmetry. The pyramidality indices, ∑(C—P—C), are 305.35 (16), 317.23 (15), 307.2 (4) and 318.67 (18)° for (I), (II), (III) and (IV), respectively. Each is found to be more pyramidal than Ph_3_P or Ph_3_PO. Hybrid DFT calculations incorporating terms for dispersion provide evidence that the causes of the increased pyramidality, despite the 3,5-dimethyl group substitution, include dispersion inter­actions. The calculated ∑(C—P—C) values are 304.8° for both (I) and (III) and 317.4° for both (II) and (IV), with no difference arising from the substitution at ring position 4.

## Abstract 2

The crystal structures of two bulky tri­aryl­phosphines of emerging inter­est in coordination chemistry and catalysis have been determined, and the syntheses and crystal structures of their corresponding oxides are reported. Each oxide is isostructural to its corresponding phosphine.

## Chemical context   

The two bulky tri­aryl­phosphines (I) and (III) are of considerable inter­est in coordination chemistry and catalysis (Kakizoe *et al.*, 2017 ▸; Lian *et al.*, 2017 ▸; Ogiwara *et al.*, 2017 ▸; Nishikawa *et al.*, 2016 ▸; Naruto *et al.*, 2015 ▸; Jover *et al.*, 2010 ▸; Romain *et al.*, 2000 ▸) and have been investigated for frustrated Lewis-pair activity (Wang & Stephan, 2014 ▸; Ullrich *et al.*, 2010 ▸). The synthesis of (I) was first mentioned in the non-patent literature by Hengartner *et al.* (1979 ▸) and in more detail twelve years later (Culcasi *et al.*, 1991 ▸) and is now commercially available from several sources, but its crystal structure has not been reported. The preparation of (III) was reported by Romain *et al.* (2000 ▸) some 11 years after it appeared in the patent literature. These authors reported a crystal structure, Cambridge Structural Database (CSD, Version 5.39, with updates to November 2017; Groom *et al.*, 2016 ▸) refcode: FOQNOO. However, as this determination used molybdenum radiation and a serial diffractometer, we have repeated it here under the same conditions as the other three compounds to improve comparability. Phosphine oxide (II) was first mentioned for its use as an additive that enhances the enanti­omeric excess in stoichiometric asymmetric epoxidation of *E*-methyl­styrene (Kerrigan *et al.*, 2002 ▸) and a schematic synthesis was reported a year later (Henschke *et al.*, 2003 ▸) but the characterization details are not found in the open literature. Similarly, phosphine oxide (IV) is mentioned only in the patent literature. Here we report the crystal structures of (I), (II) and (IV) and full details for synthesis and characterization of (II) and (IV), for the first time, and the redetermination of (III).
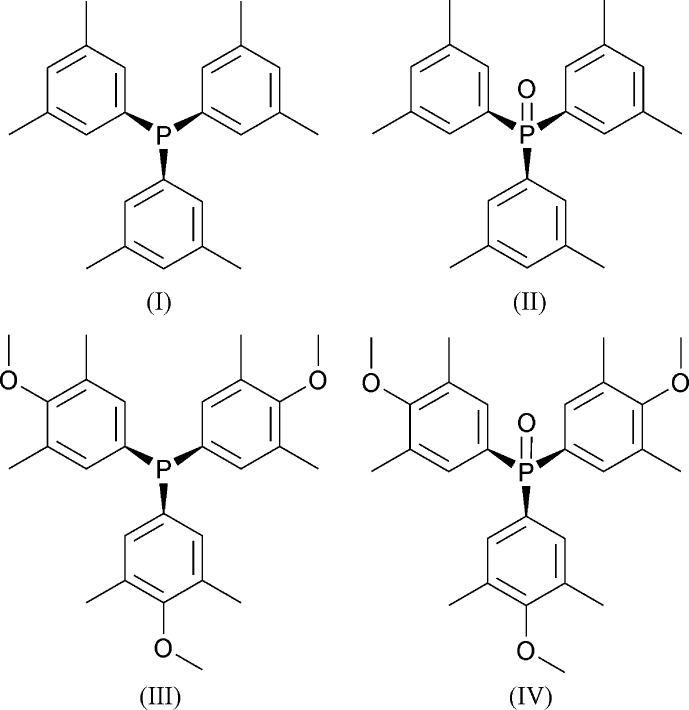


## Structural commentary   

**Figure 1:**
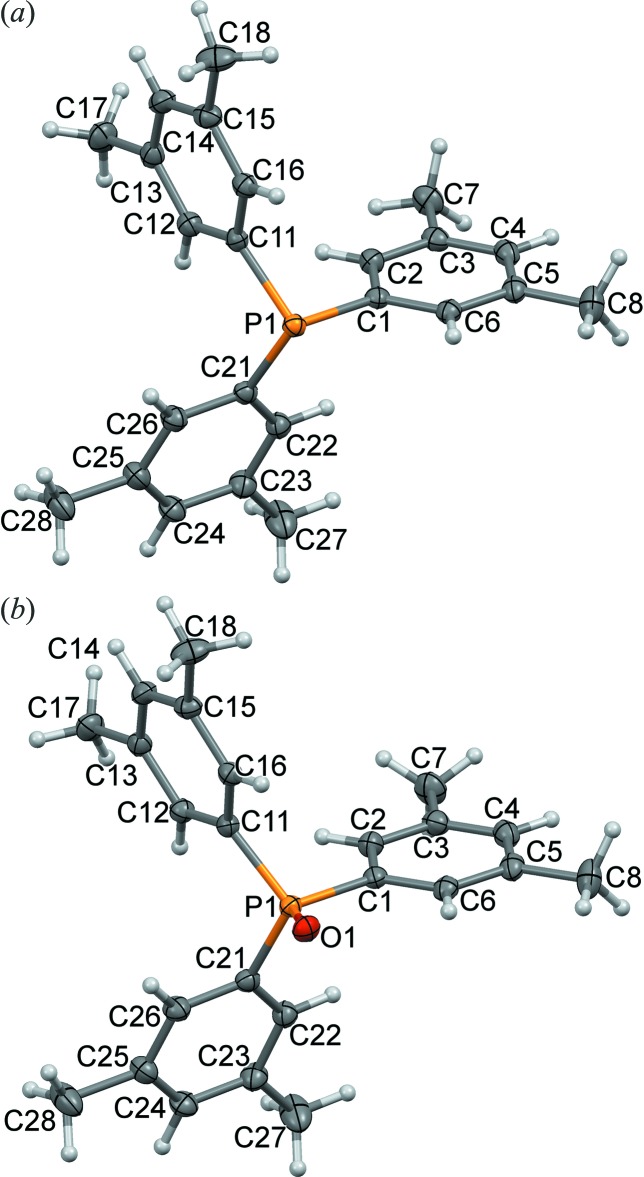
Displacement ellipsoid plots (50%) of (*a*) phosphine (I) and (*b*) phosphine oxide (II), including the atom-numbering schemes.

**Figure 2:**
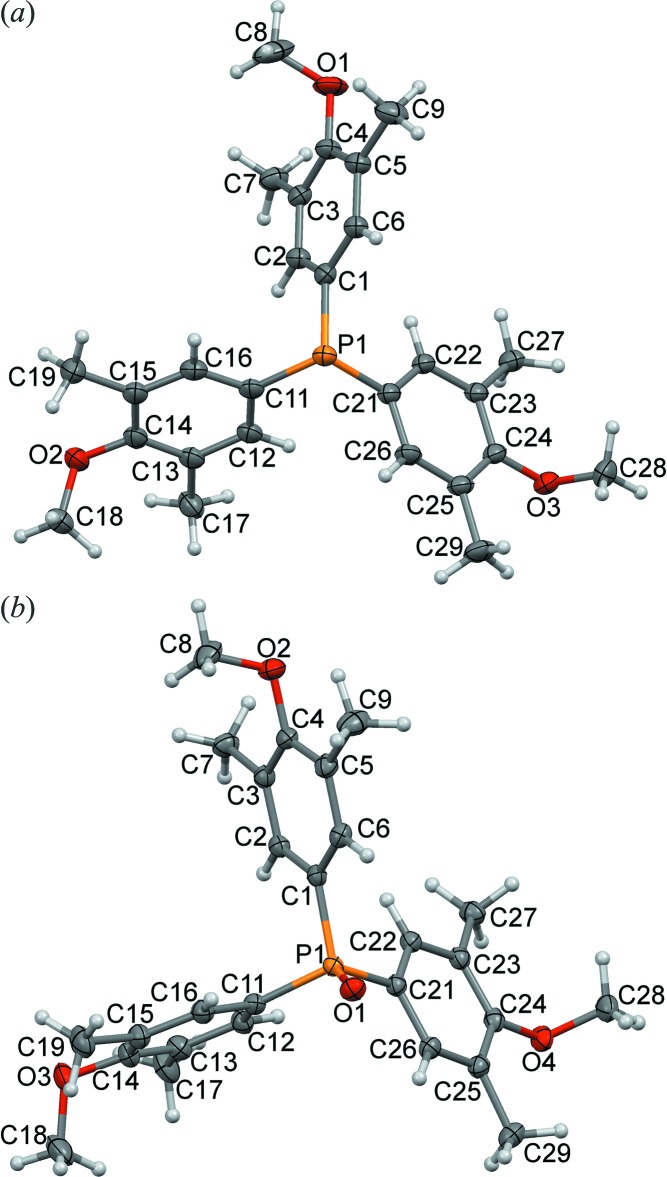
Displacement ellipsoid plots (50%) of (*a*) phosphine (III) and (*b*) phosphine oxide (IV), including the atom-numbering schemes.

Phosphine (I) crystallizes in *P*2_1_/*c* with one mol­ecule in the asymmetric unit that is distinctly pyramidal (Fig. 1 ▸). It has a sum of angles around the central phospho­rus atom, the **pyramidality index** (see Boeré & Zhang, 2013 ▸), ∑(C—P—C) = 305.35 (16)°. This is a *smaller* value than that in PPh_3_, ∑(C—P—C) = 308.3 (2)° (Boeré & Zhang, 2005 ▸), indicating a more pyramidal structure, despite the potential steric inter­ference of the three *endo*-oriented methyl substituents at C3, C13, and C23. Similarly, (III) crystallizes in *Pbca* also with *Z*′ = 1 and ∑(C—P—C) = 307.2 (4)°. By contrast, phosphines with 2,6-disubstitution patterns have greatly reduced pyramidality. For example, ∑(C—P—C) = 335.6 (3)° in Dipp_3_P, (Boeré *et al.*, 2008 ▸) 334.4 (3)° in Tripp_3_P, (Sasaki *et al.*, 2002 ▸) and 329.1 (5)° in Mes_3_P, (Blount *et al.*, 1994 ▸). Oxidation or protonation of Ar_3_P always leads to some flattening at the phospho­rus atom. Thus, although (II) is isostructural with (I), ∑(C—P—C) = 317.23 (15)° differs by some 12°, while (IV), which is isostructural with (II), has ∑(C—P—C) = 318.67 (18)° (Fig. 2 ▸). In sixteen independent structure determinations of Ph_3_PO reported in the CSD, the average value with s.u. of ∑(C—P—C) is 319.3 (3)°. Thus, for both the title phosphines and their oxides, the pyramidality index for the title compounds is lower than in the corresponding Ph_3_P or Ph_3_PO.

That all these 3,5-dimethyl-substituted compounds should be *more* pyramidal than corresponding C_6_H_5_– derivatives is at first surprising. A plausible explanation for this is that the substitution induces greater intra­molecular dispersion inter­actions, *i.e.* between the methyl groups and the π-clouds of adjacent rings. To find evidence for this, hybrid density functional theory (DFT) calculations [with Becke’s non-local three parameter exchange and the Lee–Yang–Parr correlation functional (B3LYP) and also incorporating Grimme’s D3 empirical dispersion corrections] with the 6-31G(2d,p) basis set, as implemented in the *Gaussian16* program package (Frisch *et al.*, 2016 ▸), were undertaken. The optimized geometries by DFT are characterized by common ∑(C—P—C) = 304.8° for both (I) and (III) and 317.4° for both (II) and (IV). This supports dispersion as an origin for the observed increased pyramidality caused by 3,5-dimethyl group substitution. Inter­estingly, whereas the crystal structures have flatter structures for the 4-CH_3_O derivatives (III) and (IV), the DFT calculations have identical pyramidality indices whether the substituent at the 4-position is H or CH_3_O. This indicates that inter­molecular inter­actions in the extended structures involving the meth­oxy groups affect the observed structures compared to that predicted by computation.

**Figure 3:**
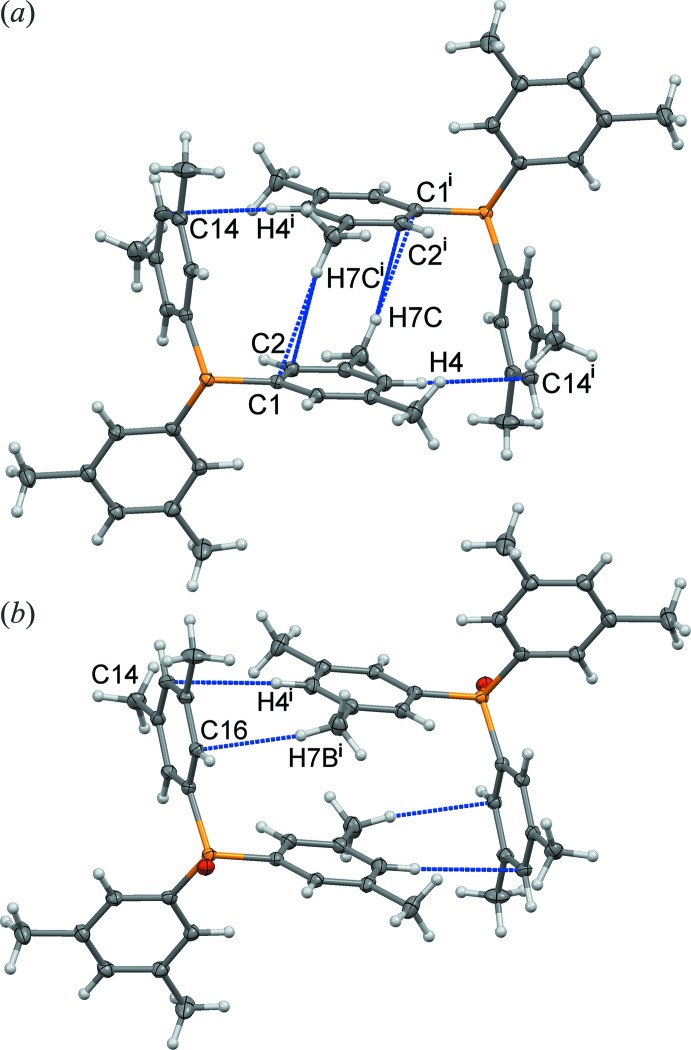
Stacking inter­actions (π–π and ‘T’ type) linking centrosymmetric pairs of (*a*) phosphine (I) and (*b*) phosphine oxide (II), which is a likely cause of the conformations adopted by the C1 rings. [Symmetry code: (i) −*x*, 1 − *y*, −*z*].

**Figure 4:**
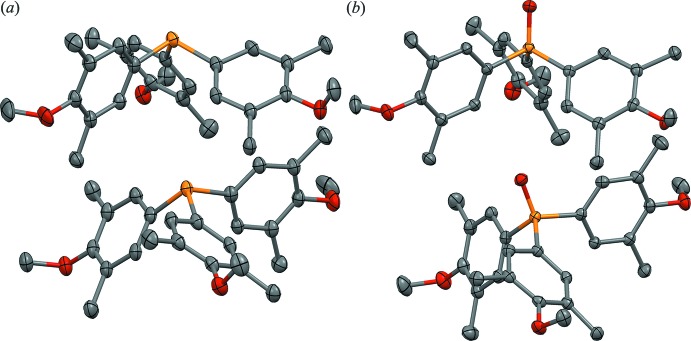
Views with the *b* axes vertical in the page, showing the staggered pyramids of (*a*) phosphine (III) and (*b*) phosphine oxide (IV) mol­ecules in their respective crystal structures. [Symmetry code for upper mol­ecules: (ii) 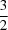 − *x*, −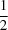 + *y*, *z*].

In the isostructural pairs, the volumes of the unit cells are larger due to oxygen incorporation. For (I) and (II), the increase is a mere 14 Å^3^ (0.7%) for the whole unit cell, or 3.5 Å^3^ per oxygen atom, whereas for (III) and (IV) the increase in volume is larger at 106 Å^3^ (2.2%) or 13.3 Å^3^ per oxygen atom. The van der Waals volume of an oxygen atom is 14.7 Å^3^. In the extended structure, the oxygen atoms in (II) are oriented into a void space (Fig. 3 ▸), whereas in (IV) they are directed towards the backside of the next P=O pyramid (Fig. 4 ▸). Thus, the nearest P⋯P^ii^ separations in the crystal increase from 5.148 (2) Å along the *b*-axis direction in (III) to 6.039 (2) Å in (IV) [Symmetry code: (ii) 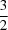 − *x*, −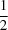 + *y*, *z*]. As a consequence, the *a*:*b* lattice parameter ratio changes from 12.30:10.27 in (III) to 11.29:11.90 in (IV).

## Supra­molecular features   

As mentioned, the supra­molecular organization in (III) and (IV) approximately stacks the Ar_3_P structures along the *b*-axis direction [the P–O vectors in (IV) alternate 21.7° off the P⋯P directions] and the rings are arranged so that alternating mol­ecules are approximately staggered (Fig. 4 ▸). This geometry facilitates helical structures, and thus the ring-tilt dihedral angles (defined from the mol­ecular threefold axis through C1,11,21 to C6,16,26) are 26.2 (1), 44.3 (1) and 49.0 (1)° in (III) and 17.0 (1), 38.8 (1) and 39.3 (1)° in (IV).

By contrast, the mol­ecules of (I) and (II) are not aligned in their crystals and are pronouncedly *less* helical in the crystals, as seen by ring-tilt dihedral angles of 35.6 (1), 8.3 (1) and 58.1 (1)° in (I) and 29.4 (1), 9.1 (1) and 61.2 (1)° in (II). In each of these structures, the C1 aryl rings are almost parallel to the mol­ecular threefold axes, a geometry that was defined as the transition state for Mislow’s ‘one-ring flip’ mechanism for racemization of propeller-shaped mol­ecules (Gust & Mislow, 1973 ▸). As shown in Fig. 3 ▸ *a*, the mol­ecules in (I) are centrosymmetrically related to one another and there are short inter­molecular contacts between the C1 rings on adjacent mol­ecules (C2 and C1 to methyl hydrogen H7*C* ^i^ of 2.84 and 2.90 Å and H4 to C14^i^ of 2.87 Å. It is likely that this packing preference is responsible for the non-helical arrangement of the rings in this structure. Similarly, in (II) short contacts link C14 with H4^i^ at 2.88 Å and C16 with methyl hydrogen H7*B* ^i^ at 2.68 Å (Fig. 3 ▸ *b*) [Symmetry code: (i) −*x*, 1 − *y*, −*z*]. There are some short inter­molecular C—H⋯O inter­actions in structures (II)–(IV), as listed in Tables 1 ▸–3 ▸ ▸.

## Database survey   

The structure of phosphine (I) can be profitably compared to six recently reported diffraction studies reported for its metal complexes or adducts. The cationic silver complex (undeca­methyl-1*H*-1-carba-closo-dodeca­borate)(tris­(3,5-di­methyl­phen­yl)phosphine)silver(I), [*L*Ag][closo-1-H-CB_11_Me_11_] (refcode ASIZIL; Clarke *et al.*, 2004 ▸) employs the large distal steric bulk from the methyl groups in (I) to hinder aggregation in the crystal. The ruthenium(II) complex (μ^2^-aqua)­bis­(μ^2^-chloro)-di­chloro­tetra­kis­[tris­(3,5-di­methyl­phen­yl)phosphine]diruthenium (COQDET01; Naruto & Saito, 2015 ▸) is part of a rational design strategy of catalysts for hydrogenation of carb­oxy­lic acids. In this complex, one ring in each unique coordinated phosphine re-orients so as to be almost orthogonal to the coordination axis, with a Ru—P—C—C torsion angles of 83.9 (3) and 87.3 (3)°. The borane complex tris­(3,5-di­methyl­phen­yl)[tris­(2,3,5,6-tetra­fluoro­phen­yl)-λ^5^-boran­yl] phospho­rane (OLAJIV; Ullrich *et al.*, 2010 ▸) is a classical rather than frustrated Lewis-pair adduct. The Tolman cone angle of (I) is estimated to be 151°. In the molybdenum complex *trans*-acetyl-dicarbon­yl(cyclo­penta­dien­yl)[tris­(3,5-di­methyl­phen­yl)phosphine]molybdenum(II) (RAHHUG; Whited *et al.*, 2017 ▸), the methyl groups on the aromatic phosphine substituents impact supra­molecular organization. The ruthenium complex di­chloro-[(*R*,*R*)-1,2-di­phenyl­ethyl­enedi­amine)­bis­[tris­(3,5-di­methyl­phen­yl)phosphine]ruthenium(II) (XARCOJ; Jing *et al.*, 2005 ▸) is competitive with chiral bidentate ligands for the enanti­oselective hydrogenation of ketones. The cationic copper complex (1,10-phenanthroline)bis­[tris­(3,5-di­methyl­phen­yl)phosphine]copper(I) tetra­fluoro­borate (BEKZOJ; Kakizoe *et al.*, 2017 ▸) is part of a study on the effects of bulky phosphines on photophysical properties of copper(I) phenanthroline complexes. Here one of the coordinated phosphines re-orients so as to have one almost orthogonal ring, with a Cu—P—C—C torsion angle of 86.6 (2)°. The structure of phosphine (III) can be compared to a single crystal structure where it is coordinated to an iridium atom that is part of an Ir_2_Mo_2_ cyclo­penta­dien­yl–carbonyl complex in tris­(μ^2^-carbon­yl)[tris­(4-meth­oxy-3,5-di­methyl­phen­yl)phos­phine]hexa­carbonyl-bis­(η^5^-cyclo­penta­dien­yl)diiridium­di­mol­yb­denum (TUTJAV; Fu *et al.*, 2016 ▸). In this complex, one of the rings is also found almost orthogonal to the coordination axis, with an Ir—P—C—C torsion angle of 73 (2)°. Thus, having one of the three aryl rings orthogonal seems to be a common configuration in crowded environments around a metal.

No crystal structures of (II) or (IV), nor any of their deriv­atives, are reported in the CSD.

## Synthesis and crystallization   

Crystals of tris­(3,5-di­methyl­phen­yl)phosphine [69227-47-0], (I), and tris­(4-meth­oxy-3,5-di­methyl­phen­yl)phosphine [121898-64-4], (III), were selected for data collection as received from Sigma–Aldrich Inc. Solvents (BDH) were chromatographic grade and used as received. NMR spectra were recorded on a 300 MHz Bruker Avance II spectrometer and are referenced to TMS at 0 (^1^H), CDCl_3_ at 77.23 (^13^C) and 85% H_3_PO_4_ at 0 ppm (capillary, ^31^P).

### Preparation of (II)   

Tris(3,5-di­methyl­phen­yl)phosphine oxide [381212-20-0], (II), was prepared by dissolving 0.10 g (I), 0.29 mmol, in 15 ml of acetone (thin-layer chromatography, TLC, monitoring: *R_f_* = 0.32 in 1:9 ethyl acetate/hexa­nes), heating to the boil, and adding 3.0 mL of 4% aqueous H_2_O_2_ dropwise. After gentle reflux for 1.5 h, the mixture was checked again by TLC (*R*_*f*_ = 0) indicating reaction completion. Removal of all volatiles, dissolving in 10 ml CH_2_Cl_2_ and drying overnight with Na_2_SO_4_, filtering and evaporating, left a dry solid. Recrystallization from mixed solvents of 5 ml heptane and 2 ml CH_2_Cl_2_ at the boil produced colourless blocks on cooling, recovered by slow evaporation to afford 0.06 g (II), 0.17 mmol, 57% yield. Identity was established by X-ray crystallography and very high purity by nuclear magnetic resonance (NMR) spec­troscopy (atom numbers are those from the C1 ring in Fig. 1 ▸ *b*). ^1^H NMR (CDCl_3_): δ 2.312 (C*H*_3_, *s*, 18H); 7.144 (C4*H*, *s*, 3H); 7.282 (C2,6*H*, *d* ^3^ *J*_PH_ = 12.3 Hz, 6H). ^13^C NMR (CDCl_3_): δ 21.47 (*C*H_3_, *s*); 129.74 (*C*2&6, *d* ^2^ *J*_PC_ = 9.8 Hz); 132.67 (*C*1, *d* ^1^ *J*_PC_ = 102.6 Hz); 133.67 (*C*4, *d* ^4^ *J*_PC_ = 3.0 Hz); 138.16 (*C*3&5, *d* ^3^ *J*_PC_ = 12.8 Hz). ^31^P NMR (CDCl_3_): δ +29.73, *s* (satellites: ^1^ *J*_PC_ = 102.6 Hz).

### Preparation of (IV)   

Tris(4-meth­oxy-3,5-di­methyl­phen­yl)phosphine oxide [540743-36-0], (IV), was similarly prepared from 0.10 g (III), 0.23 mmol, (TLC: *R_f_* = 0.38 in 1:9 ethyl acetate/hexa­nes) and 3.0 ml of 4% aqueous H_2_O_2_. 1.5 h gentle reflux also sufficed for reaction completion (TLC: *R_f_* = 0). A similar workup and recrystallization procedure afforded colourless plates by slow evaporation, 0.08 g (II), 0.18 mmol, 77% yield. Identity was established by X-ray crystallography and very high purity by nuclear magnetic resonance (NMR) spectroscopy (atom numbers are those from the C1 ring in Fig. 2 ▸ *b*). ^1^H NMR (CDCl_3_): δ 2.282 (C*H*_3_, *s*, 18H); 3.747 (C*H*_3_O, *s*, 9H); 7.311 (C2,6*H*, *d* ^3^ *J*_PH_ = 12.0 Hz, 6H). ^13^C NMR (CDCl_3_): δ 16.37 (*C*H_3_, *s*); 59.75 (*C*H_3_O, *s*); 127.84 (*C*1, *d* ^1^ *J*_PC_ = 105.7 Hz); 131.41 (*C*3&5, *d* ^3^ *J*_PC_ = 13.6 Hz); 132.81 (*C*2&6, *d* ^2^ *J*_PC_ = 10.6 Hz); 160.09 (*C*4, *d* ^4^ *J*_PC_ = 3.0 Hz). ^31^P NMR (CDCl_3_): δ +28.49, *s* (satellites: ^1^ *J*_PC_ = 105.8 Hz).

## Refinement details   

Crystal data, data collection and structure refinement details are summarized in Table 4 ▸. H atoms attached to C atoms were treated as riding, with C—H = 0.98 Å and *U*_iso_(H) = 1.5*U*_eq_(C) for methyl and C—H = 0.95 Å and *U*_iso_(H) = 1.2*U*_eq_(C) for aromatic H atoms.

## Supplementary Material

Crystal structure: contains datablock(s) I, II, III, IV. DOI: 10.1107/S2056989018007831/hb7751sup1.cif

Structure factors: contains datablock(s) I. DOI: 10.1107/S2056989018007831/hb7751Isup2.hkl

Supporting information file. DOI: 10.1107/S2056989018007831/hb7751Isup6.mol

Structure factors: contains datablock(s) II. DOI: 10.1107/S2056989018007831/hb7751IIsup3.hkl

Supporting information file. DOI: 10.1107/S2056989018007831/hb7751IIsup7.mol

Structure factors: contains datablock(s) III. DOI: 10.1107/S2056989018007831/hb7751IIIsup4.hkl

Supporting information file. DOI: 10.1107/S2056989018007831/hb7751IIIsup8.mol

Structure factors: contains datablock(s) IV. DOI: 10.1107/S2056989018007831/hb7751IVsup5.hkl

Supporting information file. DOI: 10.1107/S2056989018007831/hb7751IVsup9.mol

Supporting information file. DOI: 10.1107/S2056989018007831/hb7751Isup10.cml

Supporting information file. DOI: 10.1107/S2056989018007831/hb7751IIsup11.cml

Supporting information file. DOI: 10.1107/S2056989018007831/hb7751IIIsup12.cml

NMR data (H1, C13, P31) for compounds (II) and (IV). DOI: 10.1107/S2056989018007831/hb7751sup13.pdf

CCDC references: 1845429, 1845428, 1845427, 1845426

Additional supporting information:  crystallographic information; 3D view; checkCIF report

## Figures and Tables

**Table 1:** Hydrogen-bond geometry (Å, °) for (II)

*D*—H⋯*A*	*D*—H	H⋯*A*	*D*⋯*A*	*D*—H⋯*A*
C8—H8*A*⋯O1^i^	0.98	2.54	3.3868 (19)	144

Symmetry code: (i) 
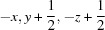
.

**Table 2:** Hydrogen-bond geometry (Å, °) for (III)

*D*—H⋯*A*	*D*—H	H⋯*A*	*D*⋯*A*	*D*—H⋯*A*
C29—H29*A*⋯O3^i^	0.98	2.58	3.524 (5)	161

Symmetry code: (i) 
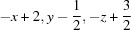
.

**Table 3:** Hydrogen-bond geometry (Å, °) for (IV)

*D*—H⋯*A*	*D*—H	H⋯*A*	*D*⋯*A*	*D*—H⋯*A*
C2—H2⋯O1^i^	0.95	2.44	3.1533 (16)	132
C7—H7*A*⋯O1^i^	0.98	2.55	3.4033 (18)	145

Symmetry code: (i) 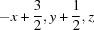.

**Table 4:** Experimental details

	(I)	(II)	(III)	(IV)
Crystal data				
Chemical formula	C_24_H_27_P	C_24_H_27_OP	C_27_H_33_O_3_P	C_27_H_33_O_4_P
*M*_r_	346.42	362.42	436.50	452.50
Crystal system, space group	Monoclinic, *P*2_1_/*c*	Monoclinic, *P*2_1_/*c*	Orthorhombic, *P* *b* *c* *a*	Orthorhombic, *P* *b* *c* *a*
Temperature (K)	108	108	109	108
*a*, *b*, *c* (Å)	14.38617 (9), 9.00514 (5), 17.22745 (12)	14.65624 (11), 8.97960 (5), 17.27940 (13)	12.3031 (6), 10.2629 (5), 37.856 (2)	11.28601 (11), 11.90008 (11), 36.3801 (3)
α, β, γ (°)	90, 112.6169 (7), 90	90, 114.2052 (9), 90	90, 90, 90	90, 90, 90
*V* (Å^3^)	2060.17 (2)	2074.16 (3)	4780.0 (4)	4886.01 (8)
*Z*	4	4	8	8
Radiation type	Cu *K*α	Cu *K*α	Cu *K*α	Cu *K*α
μ (mm^−1^)	1.18	1.23	1.21	1.24
Crystal size (mm)	0.24 × 0.2 × 0.2	0.3 × 0.2 × 0.16	0.31 × 0.07 × 0.05	0.2 × 0.2 × 0.04

Data collection				
Diffractometer	Rigaku Oxford Diffraction SuperNova, Dual, Cu at zero, Pilatus 200/300K	Rigaku Oxford Diffraction SuperNova, Dual, Cu at zero, Pilatus 200/300K	Rigaku Oxford Diffraction SuperNova, Dual, Cu at zero, Pilatus 200K	Rigaku Oxford Diffraction SuperNova, Dual, Cu at zero, Pilatus 200/300K
Absorption correction	Multi-scan (*CrysAlis PRO*; Rigaku OD, 2015 ▸)	Multi-scan (*CrysAlis PRO*; Rigaku OD, 2015 ▸)	Gaussian (*CrysAlis PRO*; Rigaku OD, 2015 ▸)	Multi-scan (*CrysAlis PRO*; Rigaku OD, 2015 ▸)
*T*_min_, *T*_max_	0.907, 1.000	0.796, 1.000	0.792, 0.950	0.755, 1.000
No. of measured, independent and observed [*I* > 2σ(*I*)] reflections	42680, 4296, 4220	48104, 4542, 4390	19900, 5029, 4084	29719, 5325, 4821
*R*_int_	0.025	0.027	0.066	0.033
(sin θ/λ)_max_ (Å^−1^)	0.630	0.641	0.640	0.639

Refinement				
*R*[*F* ^2^ > 2σ(*F* ^2^)], *wR*(*F* ^2^), *S*	0.036, 0.099, 1.05	0.037, 0.102, 1.09	0.073, 0.198, 1.05	0.039, 0.100, 1.05
No. of reflections	4296	4542	5029	5325
No. of parameters	233	242	289	299
H-atom treatment	H-atom parameters constrained	H-atom parameters constrained	H-atom parameters constrained	H-atom parameters constrained
Δρ_max_, Δρ_min_ (e Å^−3^)	0.28, −0.30	0.31, −0.30	0.54, −0.67	0.35, −0.41

Computer programs: *CrysAlis PRO* (Rigaku OD, 2015 ▸), *olex2.solve* (Bourhis *et al.*, 2015 ▸), *SHELXT* (Sheldrick, 2015*a* ▸), *SHELXL2016* (Sheldrick, 2015*b* ▸) and *OLEX2* (Dolomanov *et al.*, 2009 ▸).

## supplementary crystallographic information

### Tris(3,5-dimethylphenyl)phosphane (I) Crystal data

**Table:**

C_24_H_27_P	*F*(000) = 744
*M**_r_* = 346.42	*D*_x_ = 1.117 Mg m^−^^3^
Monoclinic, *P*2_1_/*c*	Cu *K*α radiation, λ = 1.54184 Å
*a* = 14.38617 (9) Å	Cell parameters from 33406 reflections
*b* = 9.00514 (5) Å	θ = 4.9–76.1°
*c* = 17.22745 (12) Å	µ = 1.18 mm^−^^1^
β = 112.6169 (7)°	*T* = 108 K
*V* = 2060.17 (2) Å^3^	Prism, clear colourless
*Z* = 4	0.24 × 0.2 × 0.2 mm

### Tris(3,5-dimethylphenyl)phosphane (I) Data collection

**Table:**

Rigaku Oxford Diffraction SuperNova, Dual, Cu at zero, Pilatus 200/300K diffractometer	4296 independent reflections
Radiation source: micro-focus sealed X-ray tube, SuperNova (Cu) X-ray Source	4220 reflections with *I* > 2σ(*I*)
Mirror monochromator	*R*_int_ = 0.025
ω scans	θ_max_ = 76.3°, θ_min_ = 3.3°
Absorption correction: multi-scan (CrysAlis PRO; Rigaku OD, 2015)	*h* = −18→18
*T*_min_ = 0.907, *T*_max_ = 1.000	*k* = −11→11
42680 measured reflections	*l* = −21→21

### Tris(3,5-dimethylphenyl)phosphane (I) Refinement

**Table:**

Refinement on *F*^2^	Hydrogen site location: inferred from neighbouring sites
Least-squares matrix: full	H-atom parameters constrained
*R*[*F*^2^ > 2σ(*F*^2^)] = 0.036	*w* = 1/[σ^2^(*F*_o_^2^) + (0.0521*P*)^2^ + 1.0098*P*] where *P* = (*F*_o_^2^ + 2*F*_c_^2^)/3
*wR*(*F*^2^) = 0.099	(Δ/σ)_max_ < 0.001
*S* = 1.05	Δρ_max_ = 0.28 e Å^−^^3^
4296 reflections	Δρ_min_ = −0.29 e Å^−^^3^
233 parameters	Extinction correction: SHELXL2016 (Sheldrick, 2015b), Fc^*^=kFc[1+0.001xFc^2^λ^3^/sin(2θ)]^-1/4^
0 restraints	Extinction coefficient: 0.0008 (2)
Primary atom site location: iterative	

### Tris(3,5-dimethylphenyl)phosphane (I) Special details

**Table:**

Geometry. All esds (except the esd in the dihedral angle between two l.s. planes) are estimated using the full covariance matrix. The cell esds are taken into account individually in the estimation of esds in distances, angles and torsion angles; correlations between esds in cell parameters are only used when they are defined by crystal symmetry. An approximate (isotropic) treatment of cell esds is used for estimating esds involving l.s. planes.

### Tris(3,5-dimethylphenyl)phosphane (I) Fractional atomic coordinates and isotropic or equivalent isotropic displacement parameters (Å^2^)

**Table:**

	*x*	*y*	*z*	*U*_iso_*/*U*_eq_	
P1	0.24237 (2)	0.22483 (3)	0.19991 (2)	0.01773 (10)	
C1	0.13769 (9)	0.35790 (14)	0.17356 (7)	0.0191 (2)	
C2	0.13216 (9)	0.48677 (14)	0.12664 (8)	0.0217 (3)	
H2	0.185332	0.509281	0.108761	0.026*	
C3	0.05030 (9)	0.58256 (14)	0.10568 (8)	0.0230 (3)	
C4	−0.02748 (9)	0.54669 (15)	0.13197 (8)	0.0244 (3)	
H4	−0.084271	0.610509	0.117124	0.029*	
C5	−0.02410 (9)	0.41981 (14)	0.17940 (8)	0.0236 (3)	
C6	0.05937 (9)	0.32556 (14)	0.19978 (7)	0.0207 (2)	
H6	0.062758	0.238468	0.231871	0.025*	
C7	0.04651 (11)	0.72101 (15)	0.05519 (9)	0.0301 (3)	
H7A	0.113295	0.740191	0.054603	0.045*	
H7B	0.026011	0.805557	0.080747	0.045*	
H7C	−0.002220	0.707187	−0.002560	0.045*	
C8	−0.10812 (10)	0.38474 (17)	0.20867 (10)	0.0335 (3)	
H8A	−0.114279	0.465469	0.244548	0.050*	
H8B	−0.092999	0.291750	0.240681	0.050*	
H8C	−0.171574	0.374269	0.159769	0.050*	
C11	0.25424 (9)	0.21396 (13)	0.09776 (7)	0.0188 (2)	
C12	0.30867 (9)	0.31418 (14)	0.06972 (8)	0.0205 (2)	
H12	0.346853	0.390234	0.106351	0.025*	
C13	0.30765 (9)	0.30395 (14)	−0.01141 (8)	0.0228 (3)	
C14	0.25218 (10)	0.18977 (15)	−0.06370 (8)	0.0250 (3)	
H14	0.250737	0.182438	−0.119163	0.030*	
C15	0.19901 (10)	0.08650 (14)	−0.03684 (8)	0.0246 (3)	
C16	0.20062 (9)	0.10030 (14)	0.04441 (8)	0.0209 (2)	
H16	0.164506	0.030941	0.063658	0.025*	
C17	0.36423 (11)	0.41385 (17)	−0.04288 (9)	0.0323 (3)	
H17A	0.395402	0.489222	0.000397	0.048*	
H17B	0.317411	0.461883	−0.094009	0.048*	
H17C	0.416634	0.361845	−0.055460	0.048*	
C18	0.14109 (13)	−0.03762 (17)	−0.09353 (9)	0.0374 (3)	
H18A	0.131846	−0.015172	−0.151700	0.056*	
H18B	0.075139	−0.047668	−0.089723	0.056*	
H18C	0.178614	−0.130746	−0.076018	0.056*	
C21	0.35253 (9)	0.33617 (13)	0.26264 (7)	0.0192 (2)	
C22	0.34586 (9)	0.47403 (14)	0.29659 (8)	0.0221 (3)	
H22	0.282059	0.520911	0.280729	0.027*	
C23	0.43125 (10)	0.54477 (15)	0.35355 (8)	0.0256 (3)	
C24	0.52445 (9)	0.47645 (15)	0.37487 (8)	0.0254 (3)	
H24	0.582867	0.523752	0.413673	0.031*	
C25	0.53395 (9)	0.33965 (15)	0.34040 (8)	0.0255 (3)	
C26	0.44748 (9)	0.26980 (14)	0.28504 (8)	0.0233 (3)	
H26	0.452905	0.175786	0.262120	0.028*	
C27	0.42188 (12)	0.69326 (18)	0.39067 (12)	0.0417 (4)	
H27A	0.453772	0.770149	0.369034	0.063*	
H27B	0.455274	0.688699	0.452071	0.063*	
H27C	0.350522	0.717280	0.375004	0.063*	
C28	0.63625 (11)	0.26868 (18)	0.36317 (11)	0.0396 (4)	
H28A	0.678464	0.332243	0.343964	0.059*	
H28B	0.628465	0.171292	0.336009	0.059*	
H28C	0.668170	0.256499	0.424317	0.059*	

### Tris(3,5-dimethylphenyl)phosphane (I) Atomic displacement parameters (Å^2^)

**Table:**

	*U*^11^	*U*^22^	*U*^33^	*U*^12^	*U*^13^	*U*^23^
P1	0.01727 (16)	0.01831 (17)	0.01719 (16)	−0.00102 (10)	0.00616 (12)	0.00027 (10)
C1	0.0182 (5)	0.0207 (6)	0.0169 (5)	−0.0014 (4)	0.0050 (4)	−0.0030 (4)
C2	0.0201 (5)	0.0239 (6)	0.0208 (6)	−0.0007 (5)	0.0074 (5)	−0.0003 (5)
C3	0.0233 (6)	0.0216 (6)	0.0205 (6)	0.0004 (5)	0.0045 (5)	−0.0021 (5)
C4	0.0198 (6)	0.0242 (6)	0.0265 (6)	0.0028 (5)	0.0058 (5)	−0.0052 (5)
C5	0.0208 (6)	0.0247 (6)	0.0260 (6)	−0.0023 (5)	0.0097 (5)	−0.0071 (5)
C6	0.0215 (6)	0.0209 (6)	0.0197 (6)	−0.0022 (5)	0.0081 (5)	−0.0031 (4)
C7	0.0308 (7)	0.0251 (7)	0.0318 (7)	0.0049 (5)	0.0090 (6)	0.0050 (5)
C8	0.0281 (7)	0.0311 (7)	0.0486 (9)	0.0004 (6)	0.0229 (6)	−0.0040 (6)
C11	0.0172 (5)	0.0195 (6)	0.0187 (6)	0.0030 (4)	0.0058 (4)	0.0007 (4)
C12	0.0191 (5)	0.0199 (6)	0.0218 (6)	0.0002 (5)	0.0071 (5)	−0.0005 (5)
C13	0.0215 (6)	0.0247 (6)	0.0234 (6)	0.0054 (5)	0.0098 (5)	0.0049 (5)
C14	0.0286 (6)	0.0283 (6)	0.0185 (6)	0.0074 (5)	0.0094 (5)	0.0010 (5)
C15	0.0263 (6)	0.0226 (6)	0.0213 (6)	0.0042 (5)	0.0052 (5)	−0.0021 (5)
C16	0.0200 (5)	0.0194 (6)	0.0218 (6)	0.0009 (4)	0.0064 (5)	0.0000 (4)
C17	0.0318 (7)	0.0382 (8)	0.0305 (7)	−0.0001 (6)	0.0160 (6)	0.0084 (6)
C18	0.0504 (9)	0.0310 (8)	0.0254 (7)	−0.0049 (7)	0.0086 (6)	−0.0081 (6)
C21	0.0196 (5)	0.0214 (6)	0.0165 (5)	−0.0021 (4)	0.0069 (4)	0.0007 (4)
C22	0.0201 (6)	0.0236 (6)	0.0235 (6)	−0.0008 (5)	0.0091 (5)	−0.0014 (5)
C23	0.0242 (6)	0.0246 (6)	0.0283 (6)	−0.0037 (5)	0.0106 (5)	−0.0056 (5)
C24	0.0210 (6)	0.0271 (7)	0.0257 (6)	−0.0059 (5)	0.0062 (5)	−0.0039 (5)
C25	0.0205 (6)	0.0271 (7)	0.0259 (6)	0.0001 (5)	0.0057 (5)	0.0000 (5)
C26	0.0218 (6)	0.0230 (6)	0.0230 (6)	0.0001 (5)	0.0064 (5)	−0.0023 (5)
C27	0.0296 (7)	0.0347 (8)	0.0567 (10)	−0.0047 (6)	0.0119 (7)	−0.0227 (7)
C28	0.0217 (7)	0.0366 (8)	0.0501 (9)	0.0032 (6)	0.0022 (6)	−0.0104 (7)

### Tris(3,5-dimethylphenyl)phosphane (I) Geometric parameters (Å, º)

**Table:**

P1—C1	1.8396 (12)	C14—C15	1.3915 (19)
P1—C11	1.8350 (12)	C15—C16	1.3965 (17)
P1—C21	1.8350 (12)	C15—C18	1.5071 (18)
C1—C2	1.3988 (17)	C16—H16	0.9500
C1—C6	1.3963 (16)	C17—H17A	0.9800
C2—H2	0.9500	C17—H17B	0.9800
C2—C3	1.3908 (17)	C17—H17C	0.9800
C3—C4	1.3970 (18)	C18—H18A	0.9800
C3—C7	1.5091 (18)	C18—H18B	0.9800
C4—H4	0.9500	C18—H18C	0.9800
C4—C5	1.3946 (19)	C21—C22	1.3912 (17)
C5—C6	1.4005 (17)	C21—C26	1.4023 (17)
C5—C8	1.5113 (17)	C22—H22	0.9500
C6—H6	0.9500	C22—C23	1.3967 (18)
C7—H7A	0.9800	C23—C24	1.3900 (18)
C7—H7B	0.9800	C23—C27	1.5105 (19)
C7—H7C	0.9800	C24—H24	0.9500
C8—H8A	0.9800	C24—C25	1.3969 (19)
C8—H8B	0.9800	C25—C26	1.3929 (18)
C8—H8C	0.9800	C25—C28	1.5114 (18)
C11—C12	1.3971 (17)	C26—H26	0.9500
C11—C16	1.3936 (17)	C27—H27A	0.9800
C12—H12	0.9500	C27—H27B	0.9800
C12—C13	1.3950 (17)	C27—H27C	0.9800
C13—C14	1.3974 (19)	C28—H28A	0.9800
C13—C17	1.5083 (18)	C28—H28B	0.9800
C14—H14	0.9500	C28—H28C	0.9800
			
C11—P1—C1	99.63 (5)	C16—C15—C18	120.49 (12)
C11—P1—C21	102.48 (5)	C11—C16—C15	121.20 (12)
C21—P1—C1	103.24 (5)	C11—C16—H16	119.4
C2—C1—P1	122.84 (9)	C15—C16—H16	119.4
C6—C1—P1	118.04 (9)	C13—C17—H17A	109.5
C6—C1—C2	119.09 (11)	C13—C17—H17B	109.5
C1—C2—H2	119.4	C13—C17—H17C	109.5
C3—C2—C1	121.27 (11)	H17A—C17—H17B	109.5
C3—C2—H2	119.4	H17A—C17—H17C	109.5
C2—C3—C4	118.43 (12)	H17B—C17—H17C	109.5
C2—C3—C7	120.09 (12)	C15—C18—H18A	109.5
C4—C3—C7	121.47 (12)	C15—C18—H18B	109.5
C3—C4—H4	119.1	C15—C18—H18C	109.5
C5—C4—C3	121.85 (11)	H18A—C18—H18B	109.5
C5—C4—H4	119.1	H18A—C18—H18C	109.5
C4—C5—C6	118.47 (11)	H18B—C18—H18C	109.5
C4—C5—C8	120.97 (12)	C22—C21—P1	123.48 (9)
C6—C5—C8	120.56 (12)	C22—C21—C26	118.81 (11)
C1—C6—C5	120.89 (12)	C26—C21—P1	117.28 (9)
C1—C6—H6	119.6	C21—C22—H22	119.4
C5—C6—H6	119.6	C21—C22—C23	121.21 (11)
C3—C7—H7A	109.5	C23—C22—H22	119.4
C3—C7—H7B	109.5	C22—C23—C27	120.24 (12)
C3—C7—H7C	109.5	C24—C23—C22	118.83 (12)
H7A—C7—H7B	109.5	C24—C23—C27	120.93 (12)
H7A—C7—H7C	109.5	C23—C24—H24	119.3
H7B—C7—H7C	109.5	C23—C24—C25	121.34 (12)
C5—C8—H8A	109.5	C25—C24—H24	119.3
C5—C8—H8B	109.5	C24—C25—C28	120.48 (12)
C5—C8—H8C	109.5	C26—C25—C24	118.79 (12)
H8A—C8—H8B	109.5	C26—C25—C28	120.72 (12)
H8A—C8—H8C	109.5	C21—C26—H26	119.5
H8B—C8—H8C	109.5	C25—C26—C21	120.99 (12)
C12—C11—P1	124.64 (9)	C25—C26—H26	119.5
C16—C11—P1	116.08 (9)	C23—C27—H27A	109.5
C16—C11—C12	119.22 (11)	C23—C27—H27B	109.5
C11—C12—H12	119.6	C23—C27—H27C	109.5
C13—C12—C11	120.82 (11)	H27A—C27—H27B	109.5
C13—C12—H12	119.6	H27A—C27—H27C	109.5
C12—C13—C14	118.54 (12)	H27B—C27—H27C	109.5
C12—C13—C17	121.17 (12)	C25—C28—H28A	109.5
C14—C13—C17	120.29 (12)	C25—C28—H28B	109.5
C13—C14—H14	119.1	C25—C28—H28C	109.5
C15—C14—C13	121.88 (12)	H28A—C28—H28B	109.5
C15—C14—H14	119.1	H28A—C28—H28C	109.5
C14—C15—C16	118.32 (12)	H28B—C28—H28C	109.5
C14—C15—C18	121.19 (12)		

### Tris(3,5-dimethylphenyl)(oxo)-λ5-phosphane (II) Crystal data

**Table:**

C_24_H_27_OP	*F*(000) = 776
*M**_r_* = 362.42	*D*_x_ = 1.161 Mg m^−^^3^
Monoclinic, *P*2_1_/*c*	Cu *K*α radiation, λ = 1.54184 Å
*a* = 14.65624 (11) Å	Cell parameters from 35213 reflections
*b* = 8.97960 (5) Å	θ = 5.2–80.3°
*c* = 17.27940 (13) Å	µ = 1.23 mm^−^^1^
β = 114.2052 (9)°	*T* = 108 K
*V* = 2074.16 (3) Å^3^	Prism, clear colourless
*Z* = 4	0.3 × 0.2 × 0.16 mm

### Tris(3,5-dimethylphenyl)(oxo)-λ5-phosphane (II) Data collection

**Table:**

Rigaku Oxford Diffraction SuperNova, Dual, Cu at zero, Pilatus 200/300K diffractometer	4542 independent reflections
Radiation source: micro-focus sealed X-ray tube, SuperNova (Cu) X-ray Source	4390 reflections with *I* > 2σ(*I*)
Mirror monochromator	*R*_int_ = 0.027
ω scans	θ_max_ = 81.1°, θ_min_ = 3.3°
Absorption correction: multi-scan (CrysAlis PRO; Rigaku OD, 2015)	*h* = −18→18
*T*_min_ = 0.796, *T*_max_ = 1.000	*k* = −11→11
48104 measured reflections	*l* = −22→22

### Tris(3,5-dimethylphenyl)(oxo)-λ5-phosphane (II) Refinement

**Table:**

Refinement on *F*^2^	Hydrogen site location: inferred from neighbouring sites
Least-squares matrix: full	H-atom parameters constrained
*R*[*F*^2^ > 2σ(*F*^2^)] = 0.037	*w* = 1/[σ^2^(*F*_o_^2^) + (0.0539*P*)^2^ + 0.8817*P*] where *P* = (*F*_o_^2^ + 2*F*_c_^2^)/3
*wR*(*F*^2^) = 0.102	(Δ/σ)_max_ < 0.001
*S* = 1.09	Δρ_max_ = 0.31 e Å^−^^3^
4542 reflections	Δρ_min_ = −0.29 e Å^−^^3^
242 parameters	Extinction correction: SHELXL2016 (Sheldrick, 2015b), Fc^*^=kFc[1+0.001xFc^2^λ^3^/sin(2θ)]^-1/4^
0 restraints	Extinction coefficient: 0.0012 (2)
Primary atom site location: iterative	

### Tris(3,5-dimethylphenyl)(oxo)-λ5-phosphane (II) Special details

**Table:**

Geometry. All esds (except the esd in the dihedral angle between two l.s. planes) are estimated using the full covariance matrix. The cell esds are taken into account individually in the estimation of esds in distances, angles and torsion angles; correlations between esds in cell parameters are only used when they are defined by crystal symmetry. An approximate (isotropic) treatment of cell esds is used for estimating esds involving l.s. planes.

### Tris(3,5-dimethylphenyl)(oxo)-λ5-phosphane (II) Fractional atomic coordinates and isotropic or equivalent isotropic displacement parameters (Å^2^)

**Table:**

	*x*	*y*	*z*	*U*_iso_*/*U*_eq_	
P1	0.24572 (2)	0.24916 (3)	0.18639 (2)	0.01699 (10)	
O1	0.23144 (6)	0.10379 (10)	0.22115 (5)	0.02266 (19)	
C1	0.14094 (8)	0.37373 (13)	0.16344 (7)	0.0196 (2)	
C2	0.13398 (9)	0.50699 (14)	0.11963 (7)	0.0228 (2)	
H2	0.184870	0.532338	0.101282	0.027*	
C3	0.05362 (9)	0.60297 (14)	0.10254 (8)	0.0246 (3)	
C4	−0.02072 (9)	0.56194 (15)	0.12945 (8)	0.0259 (3)	
H4	−0.076327	0.626245	0.117578	0.031*	
C5	−0.01577 (9)	0.42953 (14)	0.17318 (8)	0.0244 (3)	
C6	0.06620 (9)	0.33548 (13)	0.19023 (7)	0.0214 (2)	
H6	0.071067	0.245050	0.220211	0.026*	
C7	0.04832 (12)	0.74754 (16)	0.05675 (10)	0.0339 (3)	
H7A	0.084944	0.824684	0.097889	0.051*	
H7B	−0.021764	0.777612	0.026657	0.051*	
H7C	0.078336	0.734439	0.015891	0.051*	
C8	−0.09711 (11)	0.38991 (16)	0.20202 (10)	0.0338 (3)	
H8A	−0.106203	0.472170	0.235453	0.051*	
H8B	−0.078025	0.299696	0.236953	0.051*	
H8C	−0.159846	0.372154	0.152347	0.051*	
C11	0.25717 (8)	0.22998 (13)	0.08669 (7)	0.0181 (2)	
C12	0.30739 (8)	0.33312 (13)	0.05762 (7)	0.0198 (2)	
H12	0.342407	0.413590	0.093035	0.024*	
C13	0.30635 (9)	0.31846 (13)	−0.02321 (7)	0.0208 (2)	
C14	0.25486 (9)	0.19849 (14)	−0.07324 (7)	0.0226 (2)	
H14	0.253070	0.188474	−0.128577	0.027*	
C15	0.20594 (9)	0.09278 (13)	−0.04505 (7)	0.0224 (2)	
C16	0.20744 (8)	0.11037 (13)	0.03577 (7)	0.0195 (2)	
H16	0.174160	0.039944	0.056146	0.023*	
C17	0.35972 (10)	0.42842 (16)	−0.05602 (8)	0.0283 (3)	
H17A	0.370845	0.521303	−0.023642	0.043*	
H17B	0.318780	0.448660	−0.116113	0.043*	
H17C	0.424234	0.386794	−0.049635	0.043*	
C18	0.15251 (12)	−0.03725 (16)	−0.09981 (9)	0.0347 (3)	
H18A	0.149360	−0.023358	−0.157102	0.052*	
H18B	0.084510	−0.043764	−0.102530	0.052*	
H18C	0.188859	−0.129304	−0.075450	0.052*	
C21	0.35568 (9)	0.34982 (13)	0.25651 (7)	0.0193 (2)	
C22	0.34906 (9)	0.48414 (14)	0.29400 (8)	0.0223 (2)	
H22	0.285703	0.530544	0.278167	0.027*	
C23	0.43410 (9)	0.55168 (15)	0.35450 (8)	0.0256 (3)	
C24	0.52640 (9)	0.48300 (15)	0.37547 (8)	0.0255 (3)	
H24	0.584722	0.527923	0.416774	0.031*	
C25	0.53559 (9)	0.34976 (15)	0.33741 (8)	0.0258 (3)	
C26	0.44935 (9)	0.28327 (14)	0.27831 (8)	0.0234 (2)	
H26	0.454120	0.191864	0.252540	0.028*	
C27	0.42575 (11)	0.69639 (18)	0.39538 (11)	0.0417 (4)	
H27A	0.455221	0.776861	0.374843	0.063*	
H27B	0.461488	0.687914	0.457132	0.063*	
H27C	0.355104	0.718447	0.380735	0.063*	
C28	0.63753 (11)	0.28224 (18)	0.35922 (11)	0.0401 (4)	
H28A	0.676833	0.348165	0.339455	0.060*	
H28B	0.629938	0.184983	0.331532	0.060*	
H28C	0.671967	0.269752	0.420846	0.060*	

### Tris(3,5-dimethylphenyl)(oxo)-λ5-phosphane (II) Atomic displacement parameters (Å^2^)

**Table:**

	*U*^11^	*U*^22^	*U*^33^	*U*^12^	*U*^13^	*U*^23^
P1	0.01757 (16)	0.01839 (16)	0.01570 (16)	−0.00143 (10)	0.00751 (12)	−0.00066 (9)
O1	0.0256 (4)	0.0219 (4)	0.0222 (4)	−0.0026 (3)	0.0116 (3)	0.0018 (3)
C1	0.0188 (5)	0.0227 (6)	0.0170 (5)	−0.0005 (4)	0.0070 (4)	−0.0031 (4)
C2	0.0209 (5)	0.0264 (6)	0.0214 (5)	0.0002 (5)	0.0090 (4)	0.0008 (5)
C3	0.0236 (6)	0.0258 (6)	0.0214 (6)	0.0016 (5)	0.0062 (5)	−0.0006 (5)
C4	0.0213 (6)	0.0262 (6)	0.0282 (6)	0.0028 (5)	0.0082 (5)	−0.0044 (5)
C5	0.0218 (6)	0.0257 (6)	0.0272 (6)	−0.0033 (5)	0.0115 (5)	−0.0085 (5)
C6	0.0226 (6)	0.0212 (6)	0.0215 (5)	−0.0023 (4)	0.0103 (4)	−0.0047 (4)
C7	0.0321 (7)	0.0320 (7)	0.0362 (8)	0.0079 (5)	0.0126 (6)	0.0096 (5)
C8	0.0313 (7)	0.0284 (7)	0.0517 (9)	−0.0016 (5)	0.0271 (6)	−0.0074 (6)
C11	0.0169 (5)	0.0198 (5)	0.0176 (5)	0.0014 (4)	0.0071 (4)	−0.0001 (4)
C12	0.0194 (5)	0.0201 (5)	0.0195 (5)	−0.0006 (4)	0.0074 (4)	−0.0005 (4)
C13	0.0194 (5)	0.0231 (6)	0.0200 (5)	0.0027 (4)	0.0084 (4)	0.0033 (4)
C14	0.0251 (6)	0.0265 (6)	0.0172 (5)	0.0038 (5)	0.0095 (4)	0.0001 (5)
C15	0.0243 (6)	0.0211 (6)	0.0194 (5)	0.0015 (4)	0.0066 (4)	−0.0017 (4)
C16	0.0193 (5)	0.0188 (5)	0.0198 (5)	0.0005 (4)	0.0072 (4)	0.0006 (4)
C17	0.0286 (6)	0.0343 (7)	0.0244 (6)	−0.0028 (5)	0.0131 (5)	0.0059 (5)
C18	0.0493 (8)	0.0285 (7)	0.0238 (6)	−0.0088 (6)	0.0124 (6)	−0.0073 (5)
C21	0.0211 (5)	0.0208 (5)	0.0163 (5)	−0.0023 (4)	0.0080 (4)	0.0000 (4)
C22	0.0209 (5)	0.0230 (6)	0.0241 (6)	−0.0013 (4)	0.0104 (5)	−0.0023 (5)
C23	0.0253 (6)	0.0253 (6)	0.0276 (6)	−0.0044 (5)	0.0121 (5)	−0.0071 (5)
C24	0.0223 (6)	0.0268 (6)	0.0245 (6)	−0.0060 (5)	0.0068 (5)	−0.0048 (5)
C25	0.0214 (6)	0.0261 (6)	0.0258 (6)	−0.0003 (5)	0.0056 (5)	−0.0010 (5)
C26	0.0233 (6)	0.0221 (5)	0.0227 (6)	0.0000 (5)	0.0072 (5)	−0.0039 (5)
C27	0.0298 (7)	0.0370 (8)	0.0556 (10)	−0.0064 (6)	0.0148 (7)	−0.0250 (7)
C28	0.0233 (7)	0.0365 (8)	0.0482 (9)	0.0045 (6)	0.0023 (6)	−0.0119 (7)

### Tris(3,5-dimethylphenyl)(oxo)-λ5-phosphane (II) Geometric parameters (Å, º)

**Table:**

P1—O1	1.4872 (9)	C14—C15	1.3925 (17)
P1—C1	1.8077 (12)	C15—C16	1.3966 (16)
P1—C11	1.8063 (12)	C15—C18	1.5047 (17)
P1—C21	1.8113 (12)	C16—H16	0.9500
C1—C2	1.3971 (17)	C17—H17A	0.9800
C1—C6	1.3957 (16)	C17—H17B	0.9800
C2—H2	0.9500	C17—H17C	0.9800
C2—C3	1.3900 (17)	C18—H18A	0.9800
C3—C4	1.3981 (18)	C18—H18B	0.9800
C3—C7	1.5057 (18)	C18—H18C	0.9800
C4—H4	0.9500	C21—C22	1.3912 (16)
C4—C5	1.3947 (19)	C21—C26	1.3999 (17)
C5—C6	1.3975 (17)	C22—H22	0.9500
C5—C8	1.5109 (17)	C22—C23	1.3949 (17)
C6—H6	0.9500	C23—C24	1.3927 (18)
C7—H7A	0.9800	C23—C27	1.5077 (18)
C7—H7B	0.9800	C24—H24	0.9500
C7—H7C	0.9800	C24—C25	1.3982 (18)
C8—H8A	0.9800	C25—C26	1.3922 (17)
C8—H8B	0.9800	C25—C28	1.5100 (18)
C8—H8C	0.9800	C26—H26	0.9500
C11—C12	1.3982 (16)	C27—H27A	0.9800
C11—C16	1.3904 (16)	C27—H27B	0.9800
C12—H12	0.9500	C27—H27C	0.9800
C12—C13	1.3967 (16)	C28—H28A	0.9800
C13—C14	1.3938 (17)	C28—H28B	0.9800
C13—C17	1.5072 (16)	C28—H28C	0.9800
C14—H14	0.9500		
			
O1—P1—C1	112.59 (5)	C14—C15—C16	118.26 (11)
O1—P1—C11	112.64 (5)	C14—C15—C18	121.25 (11)
O1—P1—C21	113.69 (5)	C16—C15—C18	120.49 (11)
C1—P1—C21	106.31 (5)	C11—C16—C15	120.62 (11)
C11—P1—C1	104.63 (5)	C11—C16—H16	119.7
C11—P1—C21	106.29 (5)	C15—C16—H16	119.7
C2—C1—P1	121.03 (9)	C13—C17—H17A	109.5
C6—C1—P1	119.12 (9)	C13—C17—H17B	109.5
C6—C1—C2	119.85 (11)	C13—C17—H17C	109.5
C1—C2—H2	119.6	H17A—C17—H17B	109.5
C3—C2—C1	120.89 (11)	H17A—C17—H17C	109.5
C3—C2—H2	119.6	H17B—C17—H17C	109.5
C2—C3—C4	118.29 (12)	C15—C18—H18A	109.5
C2—C3—C7	120.19 (12)	C15—C18—H18B	109.5
C4—C3—C7	121.51 (12)	C15—C18—H18C	109.5
C3—C4—H4	119.0	H18A—C18—H18B	109.5
C5—C4—C3	122.03 (11)	H18A—C18—H18C	109.5
C5—C4—H4	119.0	H18B—C18—H18C	109.5
C4—C5—C6	118.60 (11)	C22—C21—P1	122.10 (9)
C4—C5—C8	120.44 (12)	C22—C21—C26	119.52 (11)
C6—C5—C8	120.96 (12)	C26—C21—P1	118.19 (9)
C1—C6—C5	120.34 (11)	C21—C22—H22	119.5
C1—C6—H6	119.8	C21—C22—C23	120.97 (11)
C5—C6—H6	119.8	C23—C22—H22	119.5
C3—C7—H7A	109.5	C22—C23—C27	120.44 (12)
C3—C7—H7B	109.5	C24—C23—C22	118.46 (12)
C3—C7—H7C	109.5	C24—C23—C27	121.10 (12)
H7A—C7—H7B	109.5	C23—C24—H24	119.1
H7A—C7—H7C	109.5	C23—C24—C25	121.82 (11)
H7B—C7—H7C	109.5	C25—C24—H24	119.1
C5—C8—H8A	109.5	C24—C25—C28	120.16 (12)
C5—C8—H8B	109.5	C26—C25—C24	118.58 (11)
C5—C8—H8C	109.5	C26—C25—C28	121.25 (12)
H8A—C8—H8B	109.5	C21—C26—H26	119.7
H8A—C8—H8C	109.5	C25—C26—C21	120.64 (12)
H8B—C8—H8C	109.5	C25—C26—H26	119.7
C12—C11—P1	123.22 (9)	C23—C27—H27A	109.5
C16—C11—P1	116.59 (9)	C23—C27—H27B	109.5
C16—C11—C12	120.06 (11)	C23—C27—H27C	109.5
C11—C12—H12	119.8	H27A—C27—H27B	109.5
C13—C12—C11	120.35 (11)	H27A—C27—H27C	109.5
C13—C12—H12	119.8	H27B—C27—H27C	109.5
C12—C13—C17	121.32 (11)	C25—C28—H28A	109.5
C14—C13—C12	118.31 (11)	C25—C28—H28B	109.5
C14—C13—C17	120.37 (11)	C25—C28—H28C	109.5
C13—C14—H14	118.8	H28A—C28—H28B	109.5
C15—C14—C13	122.38 (11)	H28A—C28—H28C	109.5
C15—C14—H14	118.8	H28B—C28—H28C	109.5

### Tris(3,5-dimethylphenyl)(oxo)-λ5-phosphane (II) Hydrogen-bond geometry (Å, º)

**Table:**

*D*—H···*A*	*D*—H	H···*A*	*D*···*A*	*D*—H···*A*
C8—H8*A*···O1^i^	0.98	2.54	3.3868 (19)	144

Symmetry code: (i) −*x*, *y*+1/2, −*z*+1/2.

### Tris(4-methoxy-3,5-dimethylphenyl)phosphane (III) Crystal data

**Table:**

C_27_H_33_O_3_P	*D*_x_ = 1.213 Mg m^−^^3^
*M**_r_* = 436.50	Cu *K*α radiation, λ = 1.54184 Å
Orthorhombic, *P**b**c**a*	Cell parameters from 6866 reflections
*a* = 12.3031 (6) Å	θ = 4.3–78.8°
*b* = 10.2629 (5) Å	µ = 1.21 mm^−^^1^
*c* = 37.856 (2) Å	*T* = 109 K
*V* = 4780.0 (4) Å^3^	Plate, clear colourless
*Z* = 8	0.31 × 0.07 × 0.05 mm
*F*(000) = 1872	

### Tris(4-methoxy-3,5-dimethylphenyl)phosphane (III) Data collection

**Table:**

Rigaku Oxford Diffraction SuperNova, Dual, Cu at zero, Pilatus 200K diffractometer	5029 independent reflections
Radiation source: micro-focus sealed X-ray tube, SuperNova (Cu) X-ray Source	4084 reflections with *I* > 2σ(*I*)
Mirror monochromator	*R*_int_ = 0.066
ω scans	θ_max_ = 80.6°, θ_min_ = 4.7°
Absorption correction: gaussian (CrysAlis PRO; Rigaku OD, 2015)	*h* = −15→12
*T*_min_ = 0.792, *T*_max_ = 0.950	*k* = −13→12
19900 measured reflections	*l* = −48→46

### Tris(4-methoxy-3,5-dimethylphenyl)phosphane (III) Refinement

**Table:**

Refinement on *F*^2^	Primary atom site location: dual
Least-squares matrix: full	Hydrogen site location: inferred from neighbouring sites
*R*[*F*^2^ > 2σ(*F*^2^)] = 0.073	H-atom parameters constrained
*wR*(*F*^2^) = 0.198	*w* = 1/[σ^2^(*F*_o_^2^) + (0.0756*P*)^2^ + 11.1312*P*] where *P* = (*F*_o_^2^ + 2*F*_c_^2^)/3
*S* = 1.05	(Δ/σ)_max_ = 0.001
5029 reflections	Δρ_max_ = 0.54 e Å^−^^3^
289 parameters	Δρ_min_ = −0.67 e Å^−^^3^
0 restraints	

### Tris(4-methoxy-3,5-dimethylphenyl)phosphane (III) Special details

**Table:**

Geometry. All esds (except the esd in the dihedral angle between two l.s. planes) are estimated using the full covariance matrix. The cell esds are taken into account individually in the estimation of esds in distances, angles and torsion angles; correlations between esds in cell parameters are only used when they are defined by crystal symmetry. An approximate (isotropic) treatment of cell esds is used for estimating esds involving l.s. planes.

### Tris(4-methoxy-3,5-dimethylphenyl)phosphane (III) Fractional atomic coordinates and isotropic or equivalent isotropic displacement parameters (Å^2^)

**Table:**

	*x*	*y*	*z*	*U*_iso_*/*U*_eq_	
P1	0.76672 (6)	0.54686 (8)	0.62877 (2)	0.0256 (2)	
O1	0.29598 (18)	0.6985 (3)	0.63244 (7)	0.0422 (6)	
O2	0.92563 (18)	0.7706 (3)	0.49141 (6)	0.0338 (5)	
O3	0.96498 (17)	0.8338 (3)	0.75085 (6)	0.0335 (5)	
C1	0.6240 (2)	0.5994 (3)	0.62927 (7)	0.0263 (6)	
C2	0.5880 (2)	0.7150 (3)	0.61423 (8)	0.0266 (6)	
H2	0.638858	0.771017	0.603024	0.032*	
C3	0.4788 (2)	0.7505 (3)	0.61522 (8)	0.0277 (6)	
C4	0.4056 (2)	0.6640 (3)	0.63115 (8)	0.0292 (7)	
C5	0.4387 (2)	0.5500 (3)	0.64752 (8)	0.0294 (6)	
C6	0.5494 (2)	0.5181 (3)	0.64638 (8)	0.0273 (6)	
H6	0.574042	0.440235	0.657356	0.033*	
C7	0.4408 (3)	0.8787 (4)	0.60070 (9)	0.0338 (7)	
H7A	0.503947	0.933229	0.595125	0.051*	
H7B	0.395514	0.922845	0.618304	0.051*	
H7C	0.398309	0.863773	0.579192	0.051*	
C8	0.2388 (3)	0.6623 (5)	0.60101 (12)	0.0579 (12)	
H8A	0.269918	0.708578	0.580746	0.087*	
H8B	0.161910	0.685379	0.603448	0.087*	
H8C	0.245547	0.568119	0.597310	0.087*	
C9	0.3589 (3)	0.4620 (4)	0.66631 (10)	0.0417 (8)	
H9A	0.314595	0.415649	0.648830	0.062*	
H9B	0.311580	0.514465	0.681517	0.062*	
H9C	0.398694	0.398700	0.680751	0.062*	
C11	0.8173 (2)	0.6251 (3)	0.58820 (8)	0.0270 (6)	
C12	0.8840 (2)	0.7339 (3)	0.58696 (8)	0.0283 (6)	
H12	0.903538	0.775954	0.608390	0.034*	
C13	0.9232 (2)	0.7836 (3)	0.55488 (8)	0.0286 (6)	
C14	0.8920 (2)	0.7209 (3)	0.52394 (8)	0.0297 (7)	
C15	0.8245 (2)	0.6114 (4)	0.52399 (8)	0.0310 (7)	
C16	0.7885 (2)	0.5642 (3)	0.55636 (8)	0.0291 (6)	
H16	0.743434	0.489078	0.556934	0.035*	
C17	0.9947 (3)	0.9028 (4)	0.55430 (9)	0.0379 (8)	
H17A	1.071010	0.876368	0.556053	0.057*	
H17B	0.976211	0.959229	0.574308	0.057*	
H17C	0.983239	0.950390	0.532164	0.057*	
C18	1.0338 (3)	0.7319 (4)	0.48189 (9)	0.0376 (8)	
H18A	1.055911	0.777853	0.460361	0.056*	
H18B	1.035495	0.637713	0.477671	0.056*	
H18C	1.083902	0.753749	0.501123	0.056*	
C19	0.7907 (3)	0.5467 (4)	0.48979 (8)	0.0385 (8)	
H19A	0.854690	0.508952	0.478277	0.058*	
H19B	0.757642	0.611649	0.474132	0.058*	
H19C	0.737842	0.477731	0.494810	0.058*	
C21	0.8248 (2)	0.6460 (3)	0.66423 (8)	0.0264 (6)	
C22	0.7673 (2)	0.7393 (3)	0.68323 (7)	0.0261 (6)	
H22	0.695207	0.760186	0.676218	0.031*	
C23	0.8124 (2)	0.8032 (3)	0.71231 (8)	0.0266 (6)	
C24	0.9190 (2)	0.7691 (3)	0.72226 (8)	0.0269 (6)	
C25	0.9801 (2)	0.6791 (3)	0.70352 (8)	0.0296 (7)	
C26	0.9313 (2)	0.6163 (3)	0.67470 (8)	0.0292 (7)	
H26	0.971283	0.552442	0.661997	0.035*	
C27	0.7505 (3)	0.9075 (4)	0.73180 (8)	0.0315 (7)	
H27A	0.777763	0.993395	0.724809	0.047*	
H27B	0.760265	0.895847	0.757295	0.047*	
H27C	0.673034	0.901011	0.725968	0.047*	
C28	0.9540 (3)	0.7629 (4)	0.78319 (9)	0.0434 (9)	
H28A	0.877062	0.743845	0.787437	0.065*	
H28B	0.982687	0.815264	0.802728	0.065*	
H28C	0.994753	0.681096	0.781545	0.065*	
C29	1.0948 (3)	0.6435 (4)	0.71393 (10)	0.0392 (8)	
H29A	1.094164	0.561385	0.727137	0.059*	
H29B	1.125434	0.712627	0.728759	0.059*	
H29C	1.139325	0.633387	0.692632	0.059*	

### Tris(4-methoxy-3,5-dimethylphenyl)phosphane (III) Atomic displacement parameters (Å^2^)

**Table:**

	*U*^11^	*U*^22^	*U*^33^	*U*^12^	*U*^13^	*U*^23^
P1	0.0188 (4)	0.0319 (4)	0.0261 (4)	0.0016 (3)	0.0008 (3)	0.0008 (3)
O1	0.0155 (10)	0.0541 (17)	0.0568 (15)	0.0058 (11)	0.0011 (9)	0.0071 (12)
O2	0.0254 (11)	0.0470 (14)	0.0290 (11)	0.0017 (10)	0.0051 (8)	0.0047 (10)
O3	0.0246 (10)	0.0459 (14)	0.0300 (11)	−0.0056 (10)	−0.0063 (8)	0.0003 (10)
C1	0.0219 (13)	0.0353 (16)	0.0216 (12)	−0.0012 (12)	−0.0018 (10)	−0.0025 (11)
C2	0.0192 (13)	0.0343 (17)	0.0264 (13)	−0.0011 (12)	0.0005 (10)	−0.0007 (12)
C3	0.0214 (13)	0.0370 (17)	0.0247 (13)	0.0002 (12)	−0.0036 (10)	−0.0010 (12)
C4	0.0177 (13)	0.0384 (18)	0.0314 (15)	0.0027 (12)	0.0005 (11)	−0.0023 (13)
C5	0.0213 (14)	0.0367 (17)	0.0302 (14)	−0.0009 (13)	0.0013 (11)	0.0004 (12)
C6	0.0216 (14)	0.0344 (17)	0.0259 (14)	0.0001 (12)	0.0000 (10)	0.0000 (12)
C7	0.0248 (14)	0.0368 (19)	0.0399 (17)	0.0028 (14)	−0.0044 (12)	0.0039 (14)
C8	0.0264 (17)	0.066 (3)	0.081 (3)	−0.0147 (19)	−0.0197 (18)	0.019 (2)
C9	0.0253 (16)	0.045 (2)	0.054 (2)	−0.0017 (16)	0.0080 (14)	0.0120 (17)
C11	0.0175 (12)	0.0344 (17)	0.0290 (14)	0.0053 (12)	0.0005 (10)	0.0010 (12)
C12	0.0201 (13)	0.0359 (18)	0.0289 (14)	0.0036 (12)	0.0032 (11)	−0.0004 (12)
C13	0.0209 (13)	0.0348 (17)	0.0300 (14)	0.0043 (12)	0.0036 (11)	−0.0001 (12)
C14	0.0220 (14)	0.0405 (18)	0.0266 (14)	0.0075 (13)	0.0028 (11)	0.0037 (12)
C15	0.0221 (13)	0.0428 (19)	0.0281 (14)	0.0047 (14)	0.0005 (11)	−0.0003 (13)
C16	0.0196 (13)	0.0340 (18)	0.0336 (15)	−0.0009 (12)	0.0006 (11)	0.0000 (12)
C17	0.0372 (17)	0.044 (2)	0.0320 (16)	−0.0053 (16)	0.0097 (13)	−0.0028 (14)
C18	0.0281 (16)	0.051 (2)	0.0337 (16)	−0.0012 (15)	0.0088 (13)	−0.0014 (15)
C19	0.0308 (16)	0.056 (2)	0.0284 (15)	−0.0071 (16)	−0.0001 (12)	−0.0009 (15)
C21	0.0191 (13)	0.0341 (17)	0.0260 (13)	−0.0004 (12)	−0.0005 (10)	0.0043 (11)
C22	0.0172 (13)	0.0357 (17)	0.0255 (13)	−0.0010 (12)	−0.0016 (10)	0.0037 (12)
C23	0.0177 (13)	0.0347 (17)	0.0275 (14)	−0.0025 (12)	−0.0002 (10)	0.0045 (12)
C24	0.0190 (13)	0.0348 (17)	0.0268 (14)	−0.0039 (12)	−0.0043 (10)	0.0039 (12)
C25	0.0178 (13)	0.0372 (18)	0.0337 (15)	−0.0013 (12)	−0.0028 (11)	0.0061 (13)
C26	0.0188 (13)	0.0357 (18)	0.0330 (15)	0.0055 (13)	0.0009 (11)	0.0028 (12)
C27	0.0211 (13)	0.0410 (19)	0.0324 (15)	−0.0009 (13)	−0.0031 (12)	−0.0031 (13)
C28	0.0354 (17)	0.068 (3)	0.0267 (15)	−0.0023 (18)	−0.0078 (13)	0.0063 (16)
C29	0.0213 (15)	0.048 (2)	0.048 (2)	0.0043 (15)	−0.0091 (13)	0.0041 (16)

### Tris(4-methoxy-3,5-dimethylphenyl)phosphane (III) Geometric parameters (Å, º)

**Table:**

P1—C1	1.836 (3)	C13—C17	1.507 (5)
P1—C11	1.841 (3)	C14—C15	1.398 (5)
P1—C21	1.829 (3)	C15—C16	1.390 (4)
O1—C4	1.396 (4)	C15—C19	1.513 (4)
O1—C8	1.431 (5)	C16—H16	0.9500
O2—C14	1.395 (4)	C17—H17A	0.9800
O2—C18	1.435 (4)	C17—H17B	0.9800
O3—C24	1.390 (4)	C17—H17C	0.9800
O3—C28	1.431 (4)	C18—H18A	0.9800
C1—C2	1.389 (4)	C18—H18B	0.9800
C1—C6	1.400 (4)	C18—H18C	0.9800
C2—H2	0.9500	C19—H19A	0.9800
C2—C3	1.393 (4)	C19—H19B	0.9800
C3—C4	1.400 (5)	C19—H19C	0.9800
C3—C7	1.500 (5)	C21—C22	1.391 (4)
C4—C5	1.385 (5)	C21—C26	1.403 (4)
C5—C6	1.401 (4)	C22—H22	0.9500
C5—C9	1.513 (5)	C22—C23	1.397 (4)
C6—H6	0.9500	C23—C24	1.408 (4)
C7—H7A	0.9800	C23—C27	1.507 (5)
C7—H7B	0.9800	C24—C25	1.386 (5)
C7—H7C	0.9800	C25—C26	1.402 (4)
C8—H8A	0.9800	C25—C29	1.511 (4)
C8—H8B	0.9800	C26—H26	0.9500
C8—H8C	0.9800	C27—H27A	0.9800
C9—H9A	0.9800	C27—H27B	0.9800
C9—H9B	0.9800	C27—H27C	0.9800
C9—H9C	0.9800	C28—H28A	0.9800
C11—C12	1.386 (5)	C28—H28B	0.9800
C11—C16	1.403 (4)	C28—H28C	0.9800
C12—H12	0.9500	C29—H29A	0.9800
C12—C13	1.403 (4)	C29—H29B	0.9800
C13—C14	1.390 (4)	C29—H29C	0.9800
			
C1—P1—C11	101.77 (13)	C11—C16—H16	119.3
C21—P1—C1	101.66 (14)	C15—C16—C11	121.4 (3)
C21—P1—C11	103.75 (14)	C15—C16—H16	119.3
C4—O1—C8	112.3 (3)	C13—C17—H17A	109.5
C14—O2—C18	113.3 (2)	C13—C17—H17B	109.5
C24—O3—C28	112.6 (3)	C13—C17—H17C	109.5
C2—C1—P1	123.5 (2)	H17A—C17—H17B	109.5
C2—C1—C6	119.3 (3)	H17A—C17—H17C	109.5
C6—C1—P1	117.2 (2)	H17B—C17—H17C	109.5
C1—C2—H2	119.3	O2—C18—H18A	109.5
C1—C2—C3	121.4 (3)	O2—C18—H18B	109.5
C3—C2—H2	119.3	O2—C18—H18C	109.5
C2—C3—C4	117.7 (3)	H18A—C18—H18B	109.5
C2—C3—C7	121.3 (3)	H18A—C18—H18C	109.5
C4—C3—C7	120.9 (3)	H18B—C18—H18C	109.5
O1—C4—C3	118.4 (3)	C15—C19—H19A	109.5
C5—C4—O1	118.8 (3)	C15—C19—H19B	109.5
C5—C4—C3	122.6 (3)	C15—C19—H19C	109.5
C4—C5—C6	118.0 (3)	H19A—C19—H19B	109.5
C4—C5—C9	121.6 (3)	H19A—C19—H19C	109.5
C6—C5—C9	120.4 (3)	H19B—C19—H19C	109.5
C1—C6—C5	120.8 (3)	C22—C21—P1	124.3 (2)
C1—C6—H6	119.6	C22—C21—C26	118.6 (3)
C5—C6—H6	119.6	C26—C21—P1	116.8 (2)
C3—C7—H7A	109.5	C21—C22—H22	119.0
C3—C7—H7B	109.5	C21—C22—C23	121.9 (3)
C3—C7—H7C	109.5	C23—C22—H22	119.0
H7A—C7—H7B	109.5	C22—C23—C24	117.7 (3)
H7A—C7—H7C	109.5	C22—C23—C27	121.2 (3)
H7B—C7—H7C	109.5	C24—C23—C27	121.1 (3)
O1—C8—H8A	109.5	O3—C24—C23	117.9 (3)
O1—C8—H8B	109.5	C25—C24—O3	119.7 (3)
O1—C8—H8C	109.5	C25—C24—C23	122.2 (3)
H8A—C8—H8B	109.5	C24—C25—C26	118.2 (3)
H8A—C8—H8C	109.5	C24—C25—C29	122.3 (3)
H8B—C8—H8C	109.5	C26—C25—C29	119.4 (3)
C5—C9—H9A	109.5	C21—C26—H26	119.3
C5—C9—H9B	109.5	C25—C26—C21	121.3 (3)
C5—C9—H9C	109.5	C25—C26—H26	119.3
H9A—C9—H9B	109.5	C23—C27—H27A	109.5
H9A—C9—H9C	109.5	C23—C27—H27B	109.5
H9B—C9—H9C	109.5	C23—C27—H27C	109.5
C12—C11—P1	125.4 (2)	H27A—C27—H27B	109.5
C12—C11—C16	118.6 (3)	H27A—C27—H27C	109.5
C16—C11—P1	115.9 (2)	H27B—C27—H27C	109.5
C11—C12—H12	119.1	O3—C28—H28A	109.5
C11—C12—C13	121.7 (3)	O3—C28—H28B	109.5
C13—C12—H12	119.1	O3—C28—H28C	109.5
C12—C13—C17	120.6 (3)	H28A—C28—H28B	109.5
C14—C13—C12	117.8 (3)	H28A—C28—H28C	109.5
C14—C13—C17	121.6 (3)	H28B—C28—H28C	109.5
O2—C14—C15	118.1 (3)	C25—C29—H29A	109.5
C13—C14—O2	119.5 (3)	C25—C29—H29B	109.5
C13—C14—C15	122.3 (3)	C25—C29—H29C	109.5
C14—C15—C19	121.0 (3)	H29A—C29—H29B	109.5
C16—C15—C14	118.1 (3)	H29A—C29—H29C	109.5
C16—C15—C19	120.9 (3)	H29B—C29—H29C	109.5

### Tris(4-methoxy-3,5-dimethylphenyl)phosphane (III) Hydrogen-bond geometry (Å, º)

**Table:**

*D*—H···*A*	*D*—H	H···*A*	*D*···*A*	*D*—H···*A*
C29—H29*A*···O3^i^	0.98	2.58	3.524 (5)	161

Symmetry code: (i) −*x*+2, *y*−1/2, −*z*+3/2.

### Tris(4-methoxy-3,5-dimethylphenyl(oxo)-λ5-phosphane (IV) Crystal data

**Table:**

C_27_H_33_O_4_P	*D*_x_ = 1.230 Mg m^−^^3^
*M**_r_* = 452.50	Cu *K*α radiation, λ = 1.54184 Å
Orthorhombic, *P**b**c**a*	Cell parameters from 16756 reflections
*a* = 11.28601 (11) Å	θ = 4.6–80.0°
*b* = 11.90008 (11) Å	µ = 1.24 mm^−^^1^
*c* = 36.3801 (3) Å	*T* = 108 K
*V* = 4886.01 (8) Å^3^	Plate, clear colourless
*Z* = 8	0.2 × 0.2 × 0.04 mm
*F*(000) = 1936	

### Tris(4-methoxy-3,5-dimethylphenyl(oxo)-λ5-phosphane (IV) Data collection

**Table:**

Rigaku Oxford Diffraction SuperNova, Dual, Cu at zero, Pilatus 200/300K diffractometer	5325 independent reflections
Radiation source: micro-focus sealed X-ray tube, SuperNova (Cu) X-ray Source	4821 reflections with *I* > 2σ(*I*)
Mirror monochromator	*R*_int_ = 0.033
ω scans	θ_max_ = 80.3°, θ_min_ = 4.6°
Absorption correction: multi-scan (CrysAlis PRO; Rigaku OD, 2015)	*h* = −14→13
*T*_min_ = 0.755, *T*_max_ = 1.000	*k* = −10→15
29719 measured reflections	*l* = −32→46

### Tris(4-methoxy-3,5-dimethylphenyl(oxo)-λ5-phosphane (IV) Refinement

**Table:**

Refinement on *F*^2^	Hydrogen site location: inferred from neighbouring sites
Least-squares matrix: full	H-atom parameters constrained
*R*[*F*^2^ > 2σ(*F*^2^)] = 0.039	*w* = 1/[σ^2^(*F*_o_^2^) + (0.0444*P*)^2^ + 2.732*P*] where *P* = (*F*_o_^2^ + 2*F*_c_^2^)/3
*wR*(*F*^2^) = 0.100	(Δ/σ)_max_ = 0.002
*S* = 1.05	Δρ_max_ = 0.35 e Å^−^^3^
5325 reflections	Δρ_min_ = −0.40 e Å^−^^3^
299 parameters	Extinction correction: SHELXL2016 (Sheldrick, 2015b), Fc^*^=kFc[1+0.001xFc^2^λ^3^/sin(2θ)]^-1/4^
0 restraints	Extinction coefficient: 0.00025 (5)
Primary atom site location: iterative	

### Tris(4-methoxy-3,5-dimethylphenyl(oxo)-λ5-phosphane (IV) Special details

**Table:**

Geometry. All esds (except the esd in the dihedral angle between two l.s. planes) are estimated using the full covariance matrix. The cell esds are taken into account individually in the estimation of esds in distances, angles and torsion angles; correlations between esds in cell parameters are only used when they are defined by crystal symmetry. An approximate (isotropic) treatment of cell esds is used for estimating esds involving l.s. planes.

### Tris(4-methoxy-3,5-dimethylphenyl(oxo)-λ5-phosphane (IV) Fractional atomic coordinates and isotropic or equivalent isotropic displacement parameters (Å^2^)

**Table:**

	*x*	*y*	*z*	*U*_iso_*/*U*_eq_	
P1	0.79578 (3)	0.57887 (3)	0.63306 (2)	0.01782 (10)	
O1	0.82216 (9)	0.45664 (8)	0.63566 (3)	0.0241 (2)	
O2	0.27342 (9)	0.65494 (10)	0.63075 (3)	0.0305 (2)	
O3	0.93140 (10)	0.76996 (11)	0.48735 (3)	0.0350 (3)	
O4	1.03456 (9)	0.84000 (9)	0.75134 (3)	0.0258 (2)	
C1	0.63848 (12)	0.60698 (11)	0.63380 (3)	0.0186 (3)	
C2	0.59134 (12)	0.70862 (11)	0.62114 (3)	0.0194 (3)	
H2	0.643113	0.766482	0.613037	0.023*	
C3	0.46935 (12)	0.72582 (11)	0.62030 (4)	0.0209 (3)	
C4	0.39523 (12)	0.63846 (12)	0.63188 (4)	0.0220 (3)	
C5	0.43964 (12)	0.53723 (12)	0.64567 (4)	0.0239 (3)	
C6	0.56272 (12)	0.52309 (11)	0.64628 (4)	0.0209 (3)	
H6	0.595138	0.454888	0.655395	0.025*	
C7	0.41966 (13)	0.83624 (12)	0.60764 (4)	0.0275 (3)	
H7A	0.483170	0.892330	0.606736	0.041*	
H7B	0.358199	0.861138	0.624844	0.041*	
H7C	0.385164	0.827349	0.583095	0.041*	
C8	0.22242 (15)	0.62574 (16)	0.59606 (5)	0.0385 (4)	
H8A	0.260981	0.669074	0.576526	0.058*	
H8B	0.137480	0.642838	0.596364	0.058*	
H8C	0.233832	0.545291	0.591516	0.058*	
C9	0.35923 (14)	0.44588 (15)	0.65990 (5)	0.0372 (4)	
H9A	0.375698	0.432767	0.685999	0.056*	
H9B	0.373292	0.376517	0.646050	0.056*	
H9C	0.276449	0.468978	0.656900	0.056*	
C11	0.84536 (12)	0.63908 (12)	0.58997 (4)	0.0207 (3)	
C12	0.89015 (12)	0.74785 (12)	0.58695 (4)	0.0233 (3)	
H12	0.899650	0.792197	0.608462	0.028*	
C13	0.92131 (13)	0.79276 (13)	0.55278 (4)	0.0267 (3)	
C14	0.90563 (13)	0.72557 (13)	0.52171 (4)	0.0267 (3)	
C15	0.85914 (13)	0.61662 (13)	0.52372 (4)	0.0267 (3)	
C16	0.83036 (13)	0.57450 (12)	0.55830 (4)	0.0241 (3)	
H16	0.799917	0.500336	0.560367	0.029*	
C17	0.96890 (17)	0.91105 (14)	0.54979 (4)	0.0359 (4)	
H17A	1.055600	0.908816	0.548469	0.054*	
H17B	0.944584	0.954406	0.571416	0.054*	
H17C	0.937404	0.946656	0.527556	0.054*	
C18	1.05371 (17)	0.75999 (18)	0.47752 (5)	0.0439 (4)	
H18A	1.069584	0.805621	0.455644	0.066*	
H18B	1.072181	0.681140	0.472270	0.066*	
H18C	1.103135	0.786379	0.497918	0.066*	
C19	0.83794 (16)	0.54874 (16)	0.48934 (4)	0.0367 (4)	
H19A	0.789789	0.592591	0.472108	0.055*	
H19B	0.796155	0.479203	0.495677	0.055*	
H19C	0.914092	0.530334	0.477881	0.055*	
C21	0.86395 (12)	0.66018 (11)	0.66909 (3)	0.0192 (3)	
C22	0.80829 (11)	0.75201 (11)	0.68533 (4)	0.0198 (3)	
H22	0.731414	0.773357	0.677308	0.024*	
C23	0.86343 (12)	0.81313 (11)	0.71315 (4)	0.0210 (3)	
C24	0.97618 (12)	0.77906 (12)	0.72445 (4)	0.0213 (3)	
C25	1.03682 (12)	0.69022 (12)	0.70769 (4)	0.0218 (3)	
C26	0.97866 (12)	0.63058 (11)	0.68014 (4)	0.0214 (3)	
H26	1.017338	0.568957	0.668667	0.026*	
C27	0.80642 (13)	0.91664 (12)	0.72900 (4)	0.0259 (3)	
H27A	0.765918	0.897242	0.751965	0.039*	
H27B	0.748824	0.946793	0.711416	0.039*	
H27C	0.867385	0.973307	0.733934	0.039*	
C28	0.99864 (14)	0.81185 (15)	0.78793 (4)	0.0311 (3)	
H28A	0.913403	0.825199	0.790633	0.047*	
H28B	1.042074	0.858623	0.805539	0.047*	
H28C	1.015864	0.732445	0.792677	0.047*	
C29	1.16351 (13)	0.66525 (13)	0.71787 (4)	0.0280 (3)	
H29A	1.215520	0.722386	0.707106	0.042*	
H29B	1.185603	0.590996	0.708450	0.042*	
H29C	1.171810	0.666109	0.744687	0.042*	

### Tris(4-methoxy-3,5-dimethylphenyl(oxo)-λ5-phosphane (IV) Atomic displacement parameters (Å^2^)

**Table:**

	*U*^11^	*U*^22^	*U*^33^	*U*^12^	*U*^13^	*U*^23^
P1	0.01791 (16)	0.01810 (17)	0.01746 (16)	0.00087 (12)	0.00009 (11)	−0.00058 (11)
O1	0.0246 (5)	0.0209 (5)	0.0269 (5)	0.0024 (4)	−0.0006 (4)	−0.0009 (4)
O2	0.0181 (5)	0.0398 (6)	0.0336 (5)	0.0018 (4)	−0.0010 (4)	0.0048 (5)
O3	0.0380 (6)	0.0477 (7)	0.0194 (5)	−0.0067 (5)	0.0043 (4)	0.0048 (5)
O4	0.0252 (5)	0.0319 (5)	0.0203 (4)	−0.0070 (4)	−0.0027 (4)	−0.0028 (4)
C1	0.0198 (6)	0.0206 (6)	0.0154 (5)	0.0004 (5)	−0.0007 (4)	−0.0015 (4)
C2	0.0209 (6)	0.0199 (6)	0.0172 (6)	−0.0008 (5)	0.0004 (5)	−0.0003 (5)
C3	0.0229 (6)	0.0226 (6)	0.0173 (6)	0.0022 (5)	−0.0008 (5)	−0.0008 (5)
C4	0.0175 (6)	0.0290 (7)	0.0196 (6)	0.0011 (5)	−0.0015 (5)	−0.0007 (5)
C5	0.0228 (6)	0.0258 (7)	0.0233 (6)	−0.0038 (5)	−0.0025 (5)	0.0017 (5)
C6	0.0224 (6)	0.0200 (6)	0.0205 (6)	−0.0003 (5)	−0.0038 (5)	0.0007 (5)
C7	0.0237 (7)	0.0268 (7)	0.0321 (7)	0.0050 (6)	−0.0006 (6)	0.0037 (6)
C8	0.0268 (8)	0.0465 (10)	0.0423 (9)	−0.0063 (7)	−0.0138 (7)	0.0080 (8)
C9	0.0240 (7)	0.0357 (9)	0.0518 (10)	−0.0061 (6)	−0.0033 (7)	0.0145 (7)
C11	0.0179 (6)	0.0248 (6)	0.0194 (6)	0.0018 (5)	0.0004 (5)	−0.0009 (5)
C12	0.0246 (7)	0.0262 (7)	0.0192 (6)	−0.0005 (5)	0.0023 (5)	−0.0021 (5)
C13	0.0257 (7)	0.0298 (7)	0.0245 (7)	−0.0006 (6)	0.0032 (5)	0.0007 (6)
C14	0.0251 (7)	0.0379 (8)	0.0171 (6)	−0.0003 (6)	0.0027 (5)	0.0025 (6)
C15	0.0239 (7)	0.0362 (8)	0.0200 (6)	−0.0006 (6)	0.0000 (5)	−0.0033 (6)
C16	0.0225 (6)	0.0281 (7)	0.0215 (6)	−0.0009 (5)	0.0003 (5)	−0.0027 (5)
C17	0.0473 (10)	0.0324 (8)	0.0279 (7)	−0.0073 (7)	0.0089 (7)	0.0018 (6)
C18	0.0421 (9)	0.0629 (12)	0.0269 (8)	−0.0124 (9)	0.0125 (7)	−0.0025 (8)
C19	0.0417 (9)	0.0469 (10)	0.0216 (7)	−0.0088 (8)	0.0017 (6)	−0.0075 (7)
C21	0.0203 (6)	0.0204 (6)	0.0168 (6)	−0.0011 (5)	0.0002 (5)	0.0019 (5)
C22	0.0186 (6)	0.0224 (6)	0.0182 (6)	−0.0008 (5)	0.0005 (5)	0.0015 (5)
C23	0.0222 (6)	0.0229 (6)	0.0178 (6)	−0.0020 (5)	0.0017 (5)	0.0016 (5)
C24	0.0233 (6)	0.0231 (6)	0.0175 (6)	−0.0060 (5)	−0.0008 (5)	0.0013 (5)
C25	0.0206 (6)	0.0240 (6)	0.0210 (6)	−0.0016 (5)	−0.0017 (5)	0.0048 (5)
C26	0.0215 (6)	0.0217 (6)	0.0210 (6)	0.0016 (5)	−0.0005 (5)	0.0014 (5)
C27	0.0244 (7)	0.0282 (7)	0.0250 (7)	−0.0010 (6)	0.0022 (5)	−0.0057 (5)
C28	0.0300 (8)	0.0436 (9)	0.0198 (6)	−0.0032 (7)	−0.0021 (6)	−0.0019 (6)
C29	0.0236 (7)	0.0293 (7)	0.0311 (7)	0.0011 (6)	−0.0073 (6)	0.0023 (6)

### Tris(4-methoxy-3,5-dimethylphenyl(oxo)-λ5-phosphane (IV) Geometric parameters (Å, º)

**Table:**

P1—O1	1.4878 (10)	C13—C17	1.511 (2)
P1—C1	1.8066 (14)	C14—C15	1.401 (2)
P1—C11	1.8121 (14)	C15—C16	1.393 (2)
P1—C21	1.8018 (13)	C15—C19	1.508 (2)
O2—C4	1.3893 (16)	C16—H16	0.9500
O2—C8	1.430 (2)	C17—H17A	0.9800
O3—C14	1.3880 (17)	C17—H17B	0.9800
O3—C18	1.431 (2)	C17—H17C	0.9800
O4—C24	1.3844 (16)	C18—H18A	0.9800
O4—C28	1.4314 (17)	C18—H18B	0.9800
C1—C2	1.3993 (18)	C18—H18C	0.9800
C1—C6	1.3906 (19)	C19—H19A	0.9800
C2—H2	0.9500	C19—H19B	0.9800
C2—C3	1.3923 (19)	C19—H19C	0.9800
C3—C4	1.399 (2)	C21—C22	1.3921 (18)
C3—C7	1.5010 (19)	C21—C26	1.4006 (18)
C4—C5	1.398 (2)	C22—H22	0.9500
C5—C6	1.3995 (19)	C22—C23	1.3930 (19)
C5—C9	1.508 (2)	C23—C24	1.3974 (19)
C6—H6	0.9500	C23—C27	1.5046 (19)
C7—H7A	0.9800	C24—C25	1.399 (2)
C7—H7B	0.9800	C25—C26	1.3927 (19)
C7—H7C	0.9800	C25—C29	1.5066 (19)
C8—H8A	0.9800	C26—H26	0.9500
C8—H8B	0.9800	C27—H27A	0.9800
C8—H8C	0.9800	C27—H27B	0.9800
C9—H9A	0.9800	C27—H27C	0.9800
C9—H9B	0.9800	C28—H28A	0.9800
C9—H9C	0.9800	C28—H28B	0.9800
C11—C12	1.394 (2)	C28—H28C	0.9800
C11—C16	1.3952 (19)	C29—H29A	0.9800
C12—H12	0.9500	C29—H29B	0.9800
C12—C13	1.398 (2)	C29—H29C	0.9800
C13—C14	1.396 (2)		
			
O1—P1—C1	112.13 (6)	C16—C15—C19	121.30 (14)
O1—P1—C11	112.32 (6)	C11—C16—H16	119.4
O1—P1—C21	113.16 (6)	C15—C16—C11	121.29 (14)
C1—P1—C11	104.08 (6)	C15—C16—H16	119.4
C21—P1—C1	108.02 (6)	C13—C17—H17A	109.5
C21—P1—C11	106.57 (6)	C13—C17—H17B	109.5
C4—O2—C8	112.96 (12)	C13—C17—H17C	109.5
C14—O3—C18	113.31 (13)	H17A—C17—H17B	109.5
C24—O4—C28	113.56 (11)	H17A—C17—H17C	109.5
C2—C1—P1	121.92 (10)	H17B—C17—H17C	109.5
C6—C1—P1	118.43 (10)	O3—C18—H18A	109.5
C6—C1—C2	119.62 (12)	O3—C18—H18B	109.5
C1—C2—H2	119.7	O3—C18—H18C	109.5
C3—C2—C1	120.69 (12)	H18A—C18—H18B	109.5
C3—C2—H2	119.7	H18A—C18—H18C	109.5
C2—C3—C4	118.37 (12)	H18B—C18—H18C	109.5
C2—C3—C7	120.33 (13)	C15—C19—H19A	109.5
C4—C3—C7	121.29 (12)	C15—C19—H19B	109.5
O2—C4—C3	118.53 (13)	C15—C19—H19C	109.5
O2—C4—C5	119.15 (13)	H19A—C19—H19B	109.5
C5—C4—C3	122.28 (13)	H19A—C19—H19C	109.5
C4—C5—C6	117.72 (13)	H19B—C19—H19C	109.5
C4—C5—C9	121.92 (13)	C22—C21—P1	122.53 (10)
C6—C5—C9	120.35 (13)	C22—C21—C26	119.52 (12)
C1—C6—C5	121.25 (13)	C26—C21—P1	117.93 (10)
C1—C6—H6	119.4	C21—C22—H22	119.4
C5—C6—H6	119.4	C21—C22—C23	121.11 (12)
C3—C7—H7A	109.5	C23—C22—H22	119.4
C3—C7—H7B	109.5	C22—C23—C24	117.98 (12)
C3—C7—H7C	109.5	C22—C23—C27	120.98 (12)
H7A—C7—H7B	109.5	C24—C23—C27	120.94 (12)
H7A—C7—H7C	109.5	O4—C24—C23	119.30 (12)
H7B—C7—H7C	109.5	O4—C24—C25	118.09 (12)
O2—C8—H8A	109.5	C23—C24—C25	122.43 (12)
O2—C8—H8B	109.5	C24—C25—C29	120.40 (13)
O2—C8—H8C	109.5	C26—C25—C24	117.92 (12)
H8A—C8—H8B	109.5	C26—C25—C29	121.58 (13)
H8A—C8—H8C	109.5	C21—C26—H26	119.5
H8B—C8—H8C	109.5	C25—C26—C21	120.94 (13)
C5—C9—H9A	109.5	C25—C26—H26	119.5
C5—C9—H9B	109.5	C23—C27—H27A	109.5
C5—C9—H9C	109.5	C23—C27—H27B	109.5
H9A—C9—H9B	109.5	C23—C27—H27C	109.5
H9A—C9—H9C	109.5	H27A—C27—H27B	109.5
H9B—C9—H9C	109.5	H27A—C27—H27C	109.5
C12—C11—P1	123.18 (10)	H27B—C27—H27C	109.5
C12—C11—C16	119.37 (13)	O4—C28—H28A	109.5
C16—C11—P1	117.33 (11)	O4—C28—H28B	109.5
C11—C12—H12	119.5	O4—C28—H28C	109.5
C11—C12—C13	121.08 (13)	H28A—C28—H28B	109.5
C13—C12—H12	119.5	H28A—C28—H28C	109.5
C12—C13—C17	120.64 (13)	H28B—C28—H28C	109.5
C14—C13—C12	117.98 (14)	C25—C29—H29A	109.5
C14—C13—C17	121.38 (13)	C25—C29—H29B	109.5
O3—C14—C13	119.00 (14)	C25—C29—H29C	109.5
O3—C14—C15	118.55 (13)	H29A—C29—H29B	109.5
C13—C14—C15	122.38 (13)	H29A—C29—H29C	109.5
C14—C15—C19	120.79 (13)	H29B—C29—H29C	109.5
C16—C15—C14	117.89 (13)		

### Tris(4-methoxy-3,5-dimethylphenyl(oxo)-λ5-phosphane (IV) Hydrogen-bond geometry (Å, º)

**Table:**

*D*—H···*A*	*D*—H	H···*A*	*D*···*A*	*D*—H···*A*
C2—H2···O1^i^	0.95	2.44	3.1533 (16)	132
C7—H7*A*···O1^i^	0.98	2.55	3.4033 (18)	145

Symmetry code: (i) −*x*+3/2, *y*+1/2, *z*.
