# Supplementary material for: Two tris­(3,5-disubstituted phen­yl)phosphines and their isostructural PV oxides
Source: Acta Crystallogr E Crystallogr Commun. 2018 Jun 5;74(Pt 7):889–94. doi: 10.1107/S2056989018007831 (PMC6038625; doi:10.1107/S2056989018007831)
Supplement: Supplementary file 13 [file e-74-00889-sup13.pdf]

31P{1H} tris(3,5-dimethylphenyl)phosphine oxide in CDCl3 with TMS

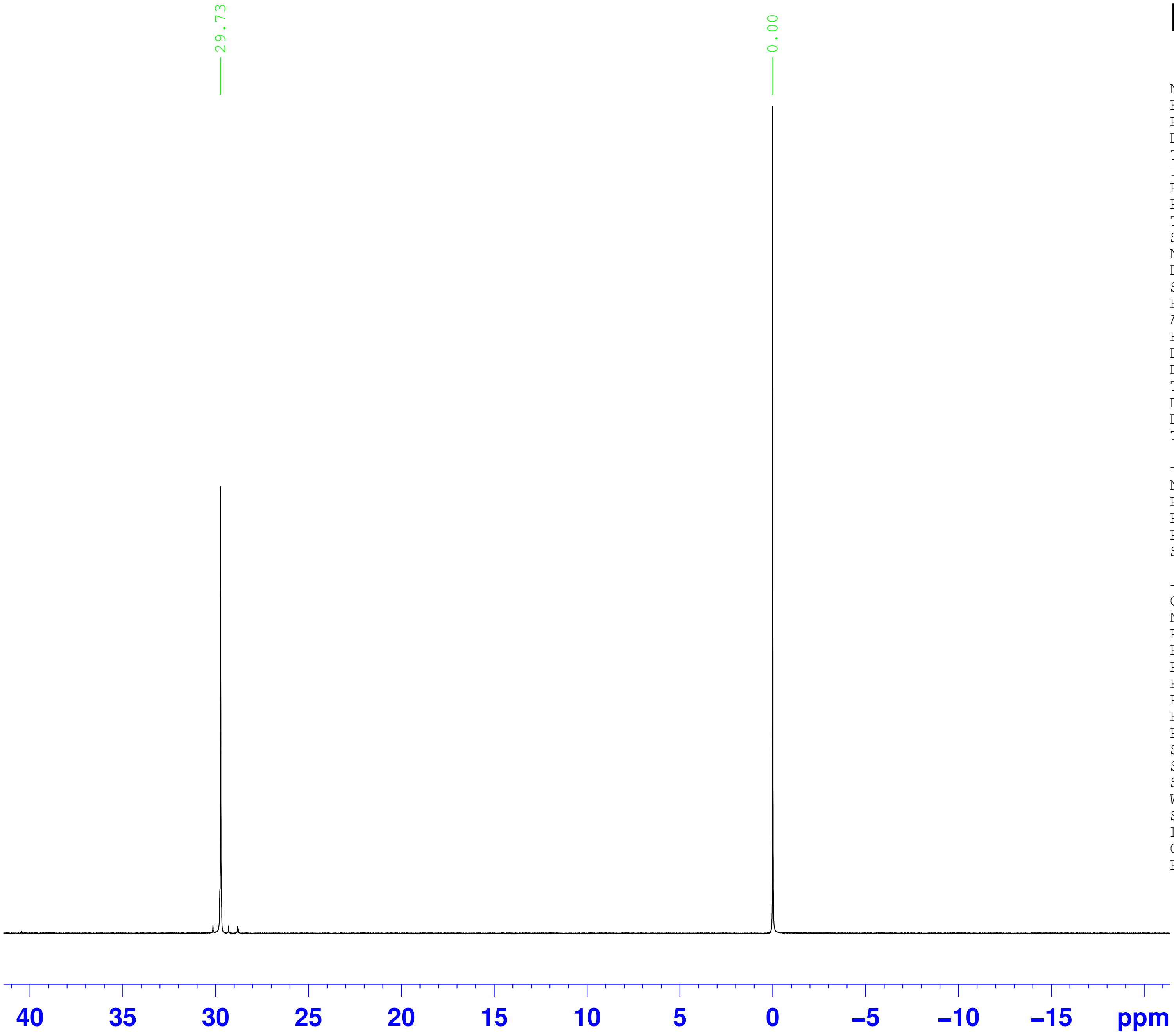

NAME	Boere Phosphine Oxides
EXPNO	6
PROCNO	1
Date_	20180510
Time	15.24
INSTRUM	spect
PROBHD	5 mm PABBO BB-
PULPROG	zgpg30
TD	131072
SOLVENT	CDCl3
NS	64
DS	4
SWH	49019.609 Hz
FIDRES	0.373990 Hz
AQ	1.3369844 sec
RG	1440
DW	10.200 usec
DE	6.00 usec
TE	292.9 K
D1	2.00000000 sec
D11	0.03000000 sec
TD0	1
======== CHANNEL f1 ========	
NUC1	31P
P1	8.80 usec
PL1	1.00 dB
PL1W	34.11898422 W
SFO1	121.4887760 MHz
======== CHANNEL f2 ========	
CPDPRG2	waltz16
NUC2	1H
PCPD2	100.00 usec
PL2	0.00 dB
PL12	18.13 dB
PL13	21.05 dB
PL2W	11.88122272 W
PL12W	0.18275161 W
PL13W	0.09329561 W
SFO2	300.1312000 MHz
SI	262144
SF	121.4948577 MHz
WDW	EM
SSB	0
LB	1.00 Hz
GB	0
PC	1.40

1H tris(3,5-dimethylphenyl)phosphine oxide in CDCl3 with TMS

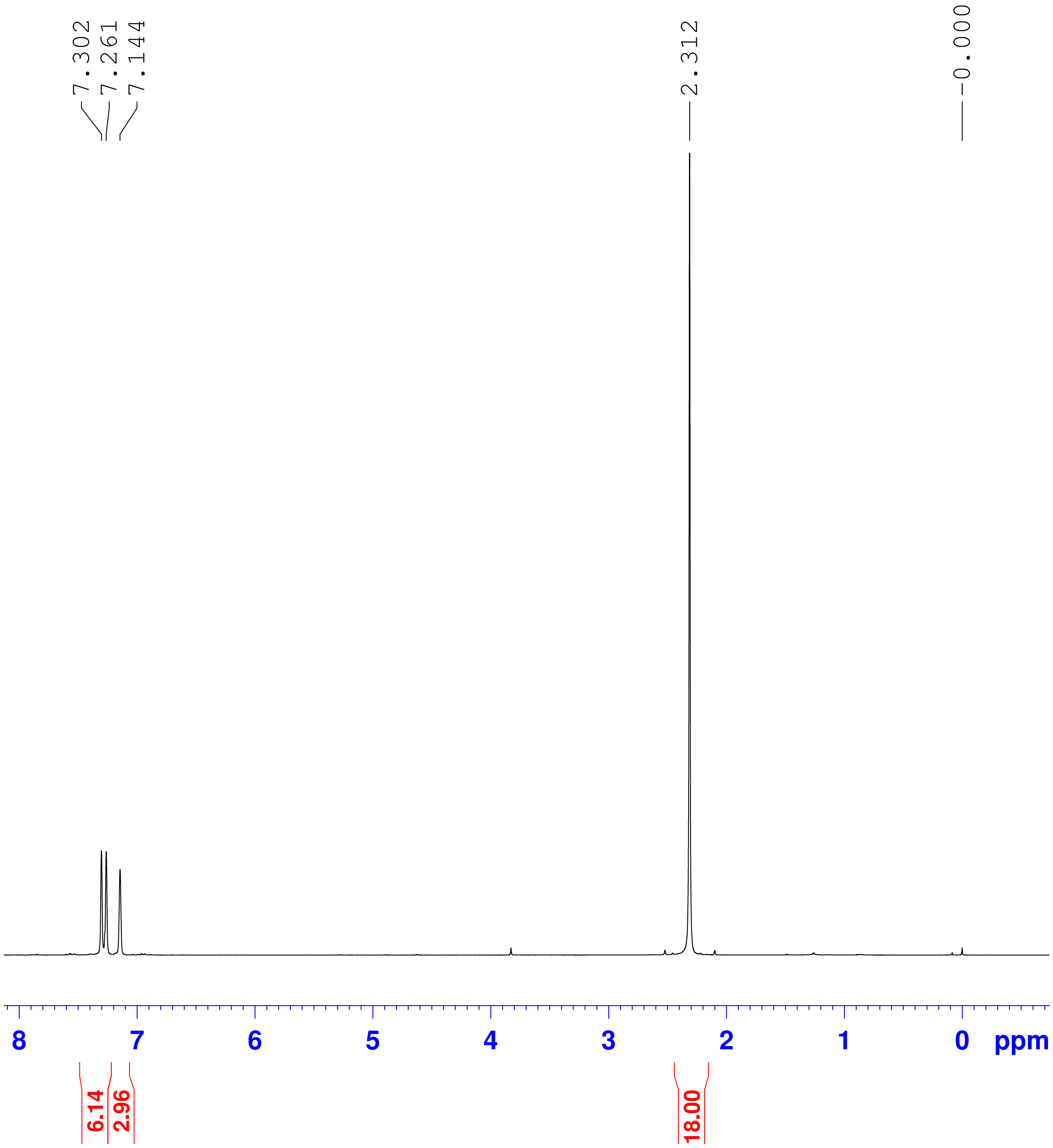

NAME	Boere Phosphine Oxide
EXPNO	2
PROCNO	1
Date_	20180504
Time	13.55
INSTRUM	spect
PROBHD	5 mm PABBO BB-
PULPROG	zg30
TD	131072
SOLVENT	CDCl3
NS	20
DS	0
SWH	6188.119 Hz
FIDRES	0.047212 Hz
AQ	10.5906677 sec
RG	32
DW	80.800 usec
DE	6.00 usec
TE	292.4 K
D1	1.00000000 sec
TD0	1

======== CHANNEL f1 ========

NUC1	1H
P1	12.40 usec
PL1	0.00 dB
PL1W	11.88122272 W
SFO1	300.1318534 MHz
SI	262144
SF	300.1300018 MHz
WDW	EM
SSB	0
LB	0.30 Hz
GB	0
PC	1.00

13C{1H} tris(3,5-dimethylphenyl)phosphine oxide in CDCl3 with TMS

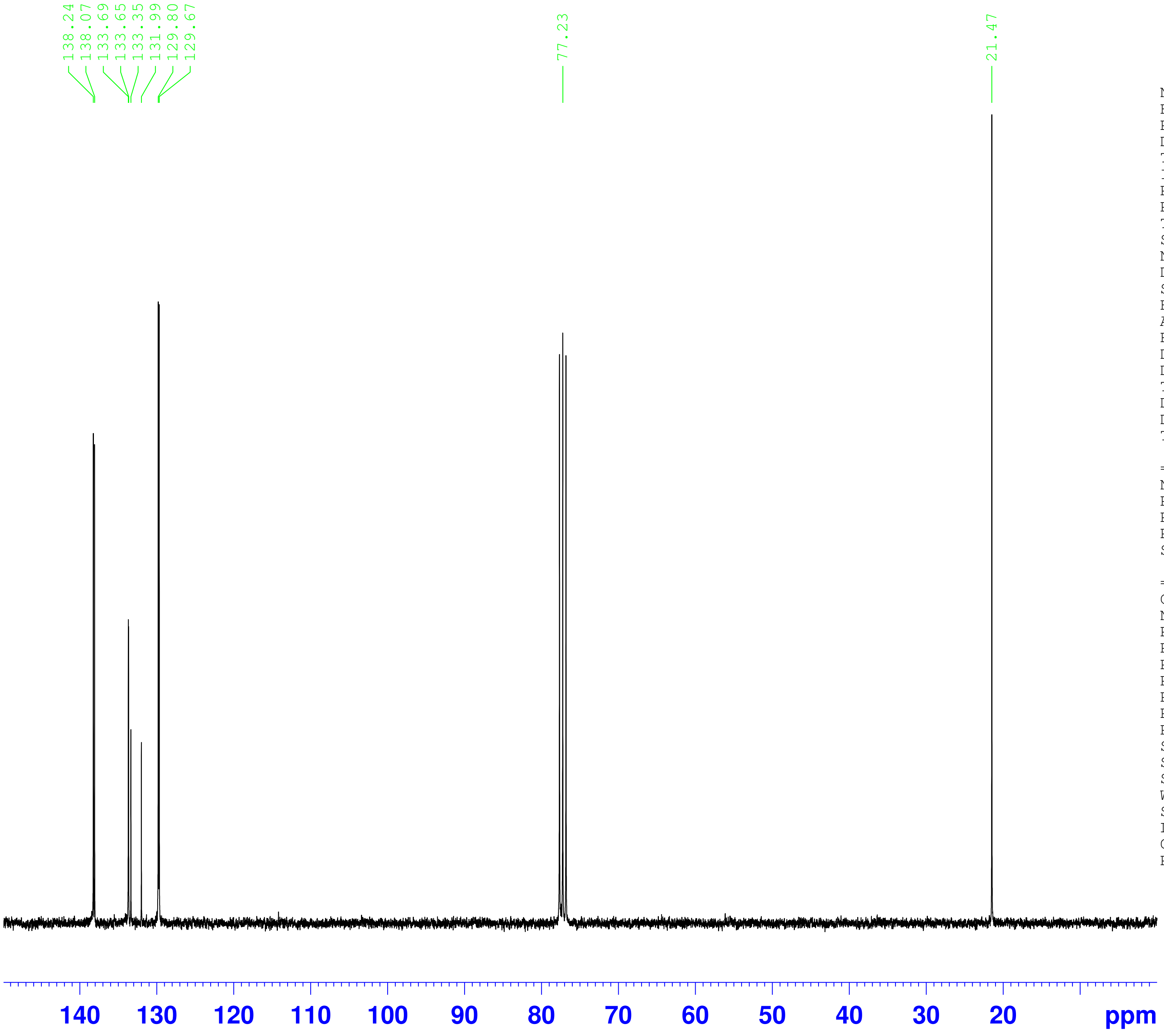

NAME	Boere Phosphine Oxides
EXPNO	4
PROCNO	1
Date_	20180504
Time	13.47
INSTRUM	spect
PROBHD	5 mm PABBO BB-
PULPROG	zgpg30
TD	131072
SOLVENT	CDCl3
NS	200
DS	2
SWH	18028.846 Hz
FIDRES	0.137549 Hz
AQ	3.6351135 sec
RG	2050
DW	27.733 usec
DE	6.00 usec
TE	292.7 K
D1	5.00000000 sec
D11	0.03000000 sec
TD0	1
======== CHANNEL f1 ========	
NUC1	13C
P1	7.60 usec
PL1	-3.00 dB
PL1W	62.14721680 W
SFO1	75.4752950 MHz
======== CHANNEL f2 ========	
CPDPRG2	waltz16
NUC2	1H
PCPD2	100.00 usec
PL2	0.00 dB
PL12	18.13 dB
PL13	21.05 dB
PL2W	11.88122272 W
PL12W	0.18275161 W
PL13W	0.09329561 W
SFO2	300.1312000 MHz
SI	262144
SF	75.4677399 MHz
WDW	EM
SSB	0
LB	1.00 Hz
GB	0
PC	1.40

31P{1H} tris(4-methoxy-3,5-dimethylphenyl)phosphine oxide in CDCl3 with TMS

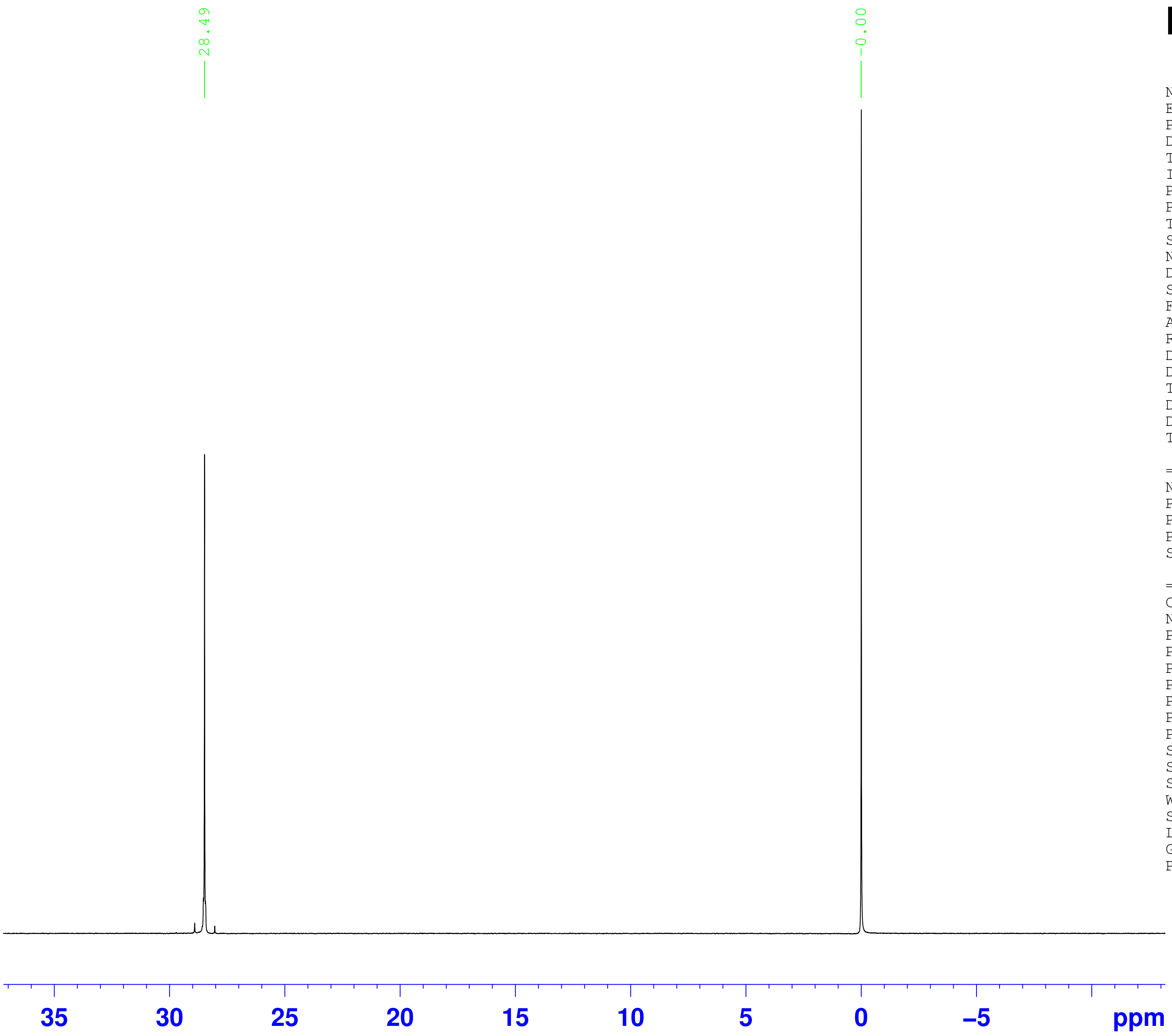

BRUKER

NAME	Boere Phosphine Oxides
EXPNO	5
PROCNO	1
Date_	20180510
Time	15.13
INSTRUM	spect
PROBHD	5 mm PABBO BB-
PULPROG	zgpg30
TD	131072
SOLVENT	CDCl3
NS	64
DS	4
SWH	49019.609 Hz
FIDRES	0.373990 Hz
AQ	1.3369844 sec
RG	1820
DW	10.200 usec
DE	6.00 usec
TE	292.9 K
D1	2.00000000 sec
D11	0.03000000 sec
TD0	1
======== CHANNEL f1 ========	
NUC1	31P
P1	8.80 usec
PL1	1.00 dB
PL1W	34.11898422 W
SFO1	121.4887760 MHz
======== CHANNEL f2 ========	
CPDPRG2	waltz16
NUC2	1H
PCPD2	100.00 usec
PL2	0.00 dB
PL12	18.13 dB
PL13	21.05 dB
PL2W	11.88122272 W
PL12W	0.18275161 W
PL13W	0.09329561 W
SFO2	300.1312000 MHz
SI	262144
SF	121.4948568 MHz
WDW	EM
SSB	0
LB	1.00 Hz
GB	0
PC	1.40

1H tris(4-methoxy-3,5-dimethylphenyl)phosphine oxide in CDCl3 with TMS

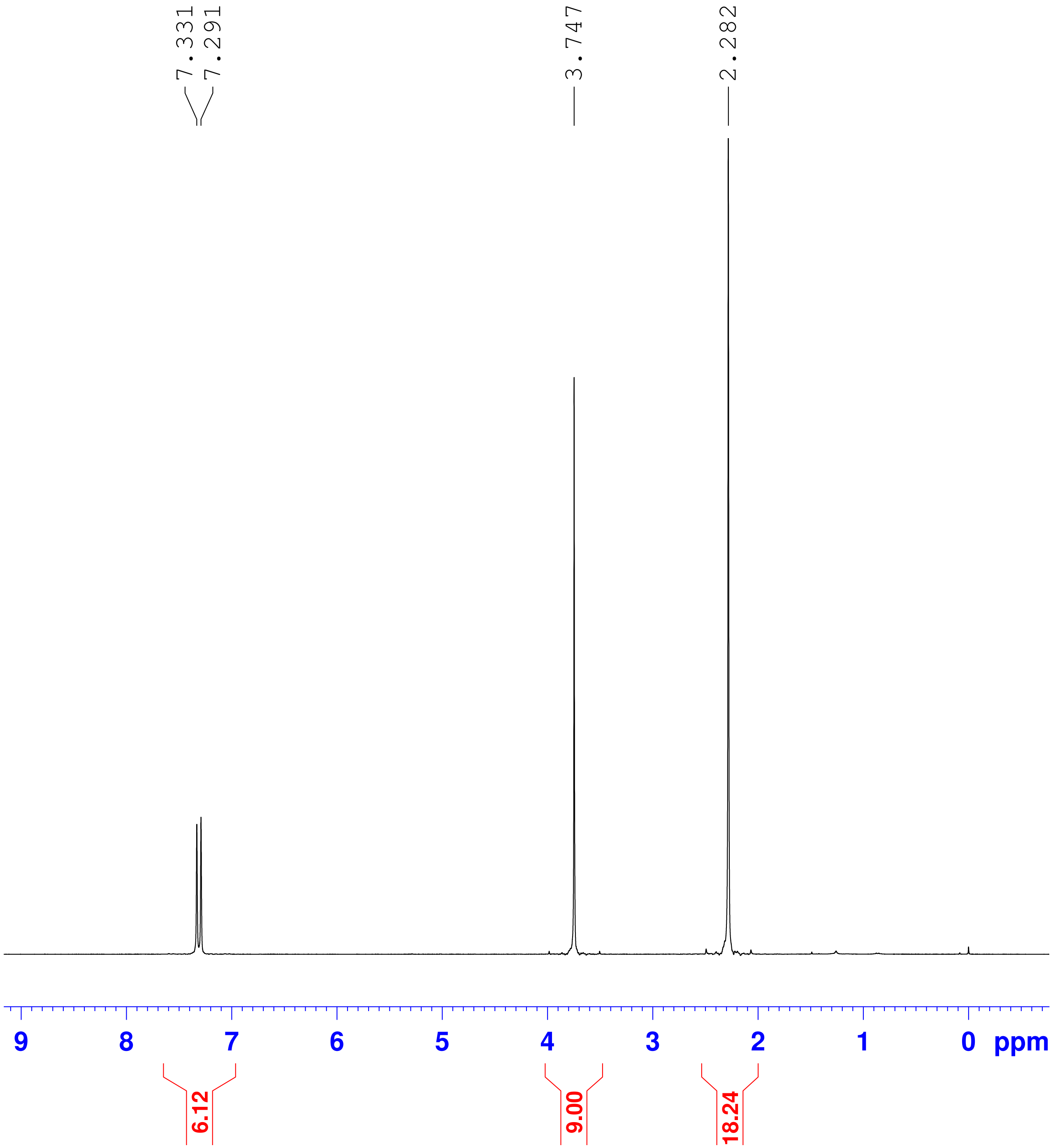

NAME	Boere Phosphine Oxide
EXPNO	1
PROCNO	1
Date_	20180504
Time	12.28
INSTRUM	spect
PROBHD	5 mm PABBO BB-
PULPROG	zg30
TD	131072
SOLVENT	C6D6
NS	20
DS	0
SWH	6188.119 Hz
FIDRES	0.047212 Hz
AQ	10.5906677 sec
RG	28.5
DW	80.800 usec
DE	6.00 usec
TE	292.3 K
D1	1.00000000 sec
TD0	1

======== CHANNEL f1 ========

NUC1	1H
P1	12.40 usec
PL1	0.00 dB
PL1W	11.88122272 W
SFO1	300.1318534 MHz
SI	262144
SF	300.1300100 MHz
WDW	EM
SSB	0
LB	0.30 Hz
GB	0
PC	1.00

13C{1H} tris(4-methoxy-3,5-dimethylphenyl)phosphine oxide in CDCl3 with TMS

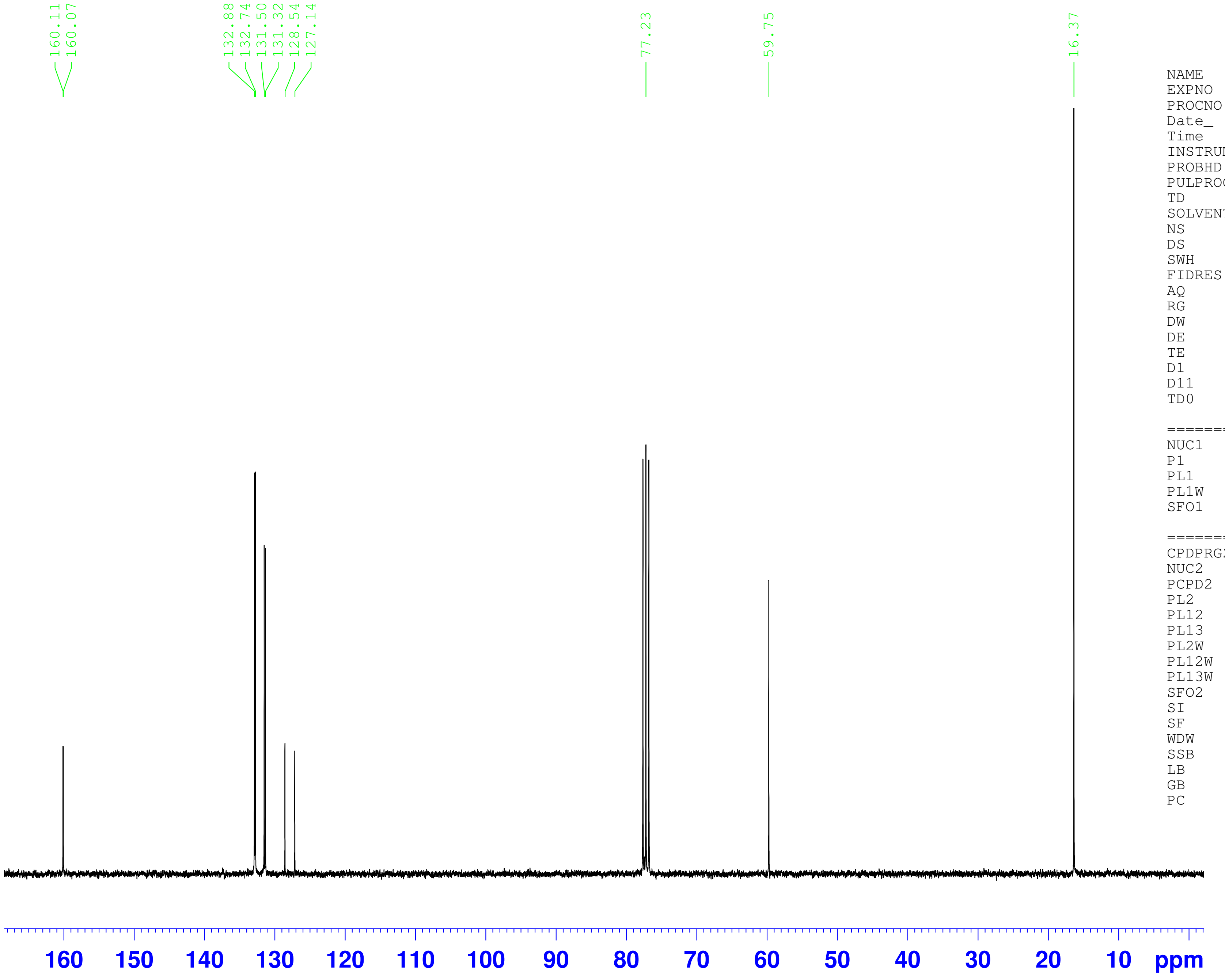

NAME	Boere Phosphine Oxides
EXPNO	3
PROCNO	1
Date_	20180504
Time	13.10
INSTRUM	spect
PROBHD	5 mm PABBO BB-
PULPROG	zgpg30
TD	131072
SOLVENT	C6D6
NS	235
DS	2
SWH	18028.846 Hz
FIDRES	0.137549 Hz
AQ	3.6351135 sec
RG	2050
DW	27.733 usec
DE	6.00 usec
TE	292.7 K
D1	5.00000000 sec
D11	0.03000000 sec
TD0	1
======== CHANNEL f1 ========	
NUC1	13C
P1	7.60 usec
PL1	-3.00 dB
PL1W	62.14721680 W
SFO1	75.4752950 MHz
======== CHANNEL f2 ========	
CPDPRG2	waltz16
NUC2	1H
PCPD2	100.00 usec
PL2	0.00 dB
PL12	18.13 dB
PL13	21.05 dB
PL2W	11.88122272 W
PL12W	0.18275161 W
PL13W	0.09329561 W
SFO2	300.1312000 MHz
SI	262144
SF	75.4677430 MHz
WDW	EM
SSB	0
LB	1.00 Hz
GB	0
PC	1.40
